# Unraveling Research Trends and Hotspots of Genetic Variants in Acute Leukemias: A Web of Science and Scopus-Based Bibliometric Study

**DOI:** 10.7150/ijms.128446

**Published:** 2026-03-30

**Authors:** Ninie Nadia Zulkipli, Nur Shafawati Ab Rajab, Nurul Aain Ahmad Fauzi, Amin Abdurrahman Abdul Rashid, Sarina Sulong

**Affiliations:** Human Genome Centre, School of Medical Sciences, Universiti Sains Malaysia, Kelantan, Malaysia.

**Keywords:** acute leukemias, bibliometric, chemotherapy, gene mutations, gene polymorphisms

## Abstract

**Background:**

Acute myeloid leukemia and acute lymphoblastic leukemia are acute leukemias frequently affecting adult and pediatric patients, respectively. Relapse and treatment resistance remain the primary limitations of standard treatments for acute leukemias. This study aimed to uncover the research trends and hotspots of acute leukemias in the past 10 years.

**Methods:**

The publications were retrieved from the Web of Science and Scopus databases, and the software tools, including Harzing's Publish or Perish, Microsoft Excel, and VOSviewer, were utilized for data analysis and visualization.

**Results:**

This study analysed 5,544 publications from 2015 to 2024, showing the fluctuating trends of publication over the years, peaking in 2021. The University of Texas System and St. Jude Children's Research Hospital were the most prolific and globally influential organizations, with 274 total publication (TP) and 1,065 total link strength (TLS), respectively. *Blood* is the top-ranked journal, featuring a total of 180 publications. Europe emerges as the top-contributor continent with robust international collaboration (TP = 3,629; TLS = 9,371). The United States becomes a top-performing country with TP, TLS, and citation values of 1,840, 1,655, and 83,210, respectively. The co-occurrence analysis of author keywords reveals gene mutations, genetic polymorphisms, and chemotherapy as the significant research hotspots.

**Conclusion:**

The research hotspots that have been identified in this study are transitioning from cytogenetic abnormalities to genomic technologies and targeted therapeutic strategies. The integration of these hotspots reflects a robust growth that emphasizes precision medicine. Further investigations that incorporate multidisciplinary approaches are necessary, as they might contribute to better management of acute leukemias.

## Introduction

Acute leukemias are malignant clonal disorders of the blood-forming organs in the hematopoietic system, which cause diffuse replacement of bone marrow with abnormal immature and undifferentiated hematopoietic cells. Unlike chronic leukemia, which progresses slowly over years, acute leukemia develops suddenly and requires immediate treatment 1. Acute myeloid leukemia (AML) results from the malignant clonal proliferation of a single myeloid progenitor cell that acquires multiple genetic and epigenetic alterations. These largely acquired alterations disrupt normal hematopoiesis by affecting critical cellular processes, including proliferation, differentiation, maturation, apoptosis, and cell cycle regulation 2, 3. *FLT3, DNMT3A*, and *NPM1* are the top three driver mutations in AML 4. While, *TP53, RUNX1, ASXL1, BCOR, EZH2, SF3B1, SRSF2, STAG2, U2AF1*, and *ZRSR2* are reported as the most frequent gene mutations. A number of new therapies, including venetoclax, oral azacytidine, and revumenib, have been approved by the US Food and Drug Administration (FDA) since 2017 5. The second type is acute lymphoblastic leukemia (ALL). It is the most common childhood cancer across the globe, and it contributes to ~80% of all childhood leukemias 6. It is also a significant contributor to cancer mortality prior to the age of 40 7. It develops from the clonal expansion of a single lymphoid progenitor cell, resultant of distinct genetic and epigenetic changes. B-cells and T-cells are the lineages of ALL. Among ALL cases in children, the B-cell acute lymphoblastic leukemia (B-ALL) accounts for ~85% of all cases of ALL and is marked by the uncontrolled growth of immature B-lymphoblasts. The most common gene mutations that are associated with B-ALL include *PAX5, TP53, BLNK, IKZF1*, and *RUNX1* 8. In 2018, the Associazione Italiana Ematologia and Oncologia Pediatrica-Berlin-Frankfurt-Muenster (AIEOP-BFM) consortium introduced *IKZF1*plus as a highly adverse biomarker in B-ALL 9. It is characterized by the *IKZF1* deletion co-occurring with *PAX5, PAR1*, or *CDKN2A/B* region deletions in the absence of *ERG* loss 10. Additionally, *IKZF1*plus classification excludes the common genetic losses that affect *EBF1, ETV6, BTG1*, and *RB1* 9. In contrast, T-cell acute lymphoblastic leukemia (T-ALL) is a more commonly aggressive type of ALL and contributes to ~15% of all ALL cases; it is twice as prevalent in males as in females 8. *NOTCH1, FBXW7, PTEN, CDKN2,* and *STAT5* are the most common gene mutations in T-ALL 11.

Telomerase is a ribonucleoprotein that comprises a catalytic telomerase reverse transcriptase subunit (TERT), and a telomerase RNA component (TERC) that are responsible for maintaining telomere length and serve as a template for the synthesis of telomeres, respectively 12, 13. Telomerase remains in an inactive-state in somatic cells but not in germ and stem cells 14. In cancer cells, telomerase is activated up to 95% 15. The alteration of telomerase function not only affected progenitor cell proliferation but also led to extremely short telomeres 16. Telomere shortening contributes to the susceptibility and progression of acute leukemias. Telomere shortening increased breakage-fusion-bridge events, leading to genome instability and structural chromosome rearrangements 17. AML patients were reported to have longer telomeres than ALL patients. Conversely, AML patients with worsening disease have shorter telomeres than ALL patients. ALL patients with complex karyotypes have higher rates of telomere shortening compared to patients with normal karyotypes. The rate of telomere shortening reflects the aggressiveness of ALL 18. Furthermore, T-ALL patients have longer telomeres than B-ALL patients. The high telomerase activity in combination with short telomere length is associated with poor prognosis of acute leukemias and implicated in relapse and advanced disease progression 19.

Currently, bibliometrics have gained significant popularity across disciplines in academic settings 20-22. It was documented that more than 30,000 bibliometrics-related publications were published in each established public database, such as Scopus and Web of Science. The significant trajectory of this type of publication from 2019 onwards. Bibliometrics offers the utmost capability to process enormous volumes of scientific data and decipher the research impact through two approaches: i) performance analysis (trend publication, and productivity of organization, journals, and authors); and ii) science mapping (bibliographic coupling, citation, co-authorship, co-citation, and co-word analyses). To date, none of the available public databases is developed for the bibliometric approach 23. Thus, the databases, including Web of Science, Scopus, Google Scholar, and PubMed, can be utilized for data acquisition. The use of more than one database offers several advantages, including providing more comprehensive coverage of scientific searches, mitigating the risk of gaps and biases, and manifesting a more accurate content of the research topic 24, 25. The approach of data acquisition is not only limited to using a single database but also multiple databases as well. Besides, a plethora of tools and software, such as Harzing's Publish or Perish, Microsoft Office, RStudio, Biblioshiny, VOSviewer, and CiteSpace, are available for data analysis and visualization. Of note, the use of two or multiple databases is a bit of a challenge, as they generate different kinds of formats for downloading bibliometric data, need to merge the output files from each database carefully and remove any duplicate publications across the databases. The steps of data cleaning and refining are crucial, as they can affect the quality of data analysis, and hence, might be reporting inaccurate findings of the study.

To the best of our knowledge, this is the first bibliometric analysis conducted on the genetic variants of both acute leukemias (AML and ALL). Thus, this study aimed to elucidate the research trends and hotspots of genetic variants in acute leukemias indexed by Web of Science and Scopus databases from 2015 to 2024.

## Methods

### Data sources and search strategy

We utilized the Clarivate Analytics Web of Science Core Collection database (WoSCC) and Scopus databases to determine and collect publications on genetic variants in acute leukemias. The search query employed in WoSCC was as follows: Topic searches included “telomerase complex genes”, “telomerase reverse transcriptase”, “TERT”, “telomerase RNA component”, “TERC”, “mutation*”, “polymorphism*”, “variant*”, “variation*”, “alteration*”, “aberration*” and “acute leuk*mia*” within the Science Citation Index Expanded (SCIE) edition. The search string applied in Scopus was a combination of queries as follows: TITLE-ABS-KEY (“telomerase complex genes AND mutation* OR variant* OR polymorphism* OR variation* OR alteration* OR aberration* AND acute leuk*mia* OR telomerase reverse transcriptase OR TERT AND telomerase RNA component OR TERC AND mutation* OR variant* OR polymorphism* OR variation* OR alteration* OR aberration* AND acute leuk*mia*”). In this study, the keywords "telomerase," "complex genes," "telomerase reverse transcriptase," "TERT," "telomerase RNA component," and "TERC" were utilized in the search string due to their genetic variants contributing to the disease pathogenesis of acute leukemias 26-29. Publications that met the following inclusion criteria were included in this study: (1) publication timeframe ranged from 2015 to 2024, (2) English-language literature, and (3) research articles and reviews. As a result, the initial search resulted in 7,389 publications. This study adapted the Preferred Reporting Items for Systematic Reviews and Meta-Analyses (PRISMA) framework 30 for the purpose of filtering and screening relevant publications.

### Data cleaning and refining

**Figure 1:**
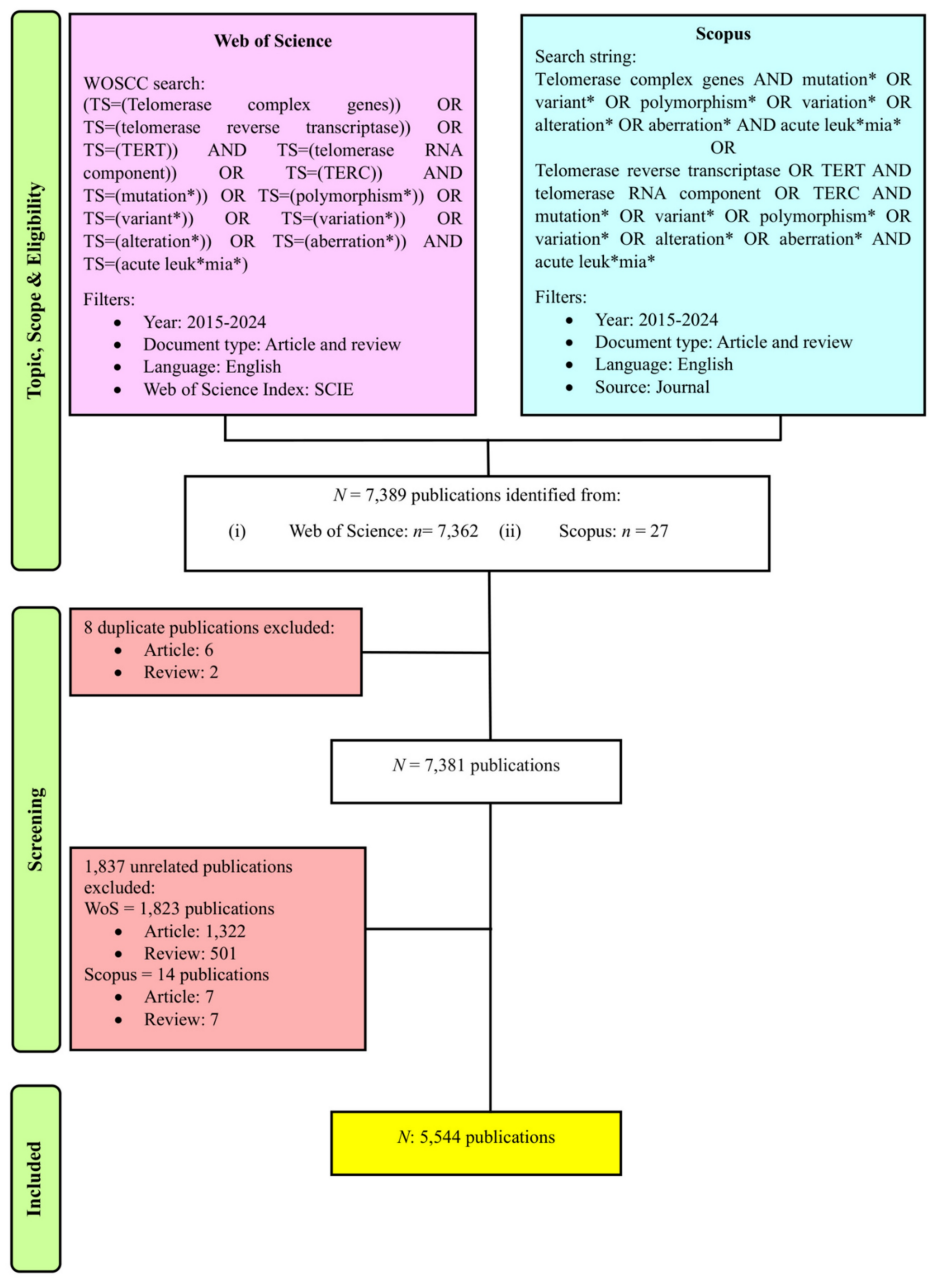
PRISMA flowchart of this study 30.

Data cleaning and refining are vital procedures where the duplicate and irrelevant publications and keywords are discarded, and metadata are standardized. This is to ensure the data are accurate and consistent and resulting in reliable, transparent, and reproducible outputs 23, 25. In this study, to mitigate bias, two independent reviewers performed the search together, and the search was completed on May 18, 2025. These two independent reviewers further refined the selection where they performed manual screening for two stages. In cases of disparities mattering rising up, this conflict will be solved at a group discussion. During the first stage of the procedure, they screened for the presence of duplicate publications across the databases. As the first stage of the procedure was passed, they manually reviewed the titles, keywords, and abstracts to select publications related to research on genetic variants in acute leukemias, resulting in 5,544 publications for subsequent analysis, as showed in Figure 1. The metadata of the selected publications from both databases were standardized and merged together, where it was necessary and relevant to do so. Subsequently completing the prior step, the metadata from each database was merged and stored in .csv and. ris format for use as input data in the software tools employed in the bibliometric analysis. Each of the input files was crucial for different analyses and used distinct software. Furthermore, in this study, we conducted data cleaning and refining particularly for three analyses: organizations, countries, and co-occurrence of author keywords. We prepared a specific thesaurus.csv file for each analysis and attached it before proceeding to data analysis and visualization steps.

### Data analysis and visualization

We utilized Microsoft Excel Office 365 for descriptive analysis and graph plotting purposes, Harzing's Publish or Perish (version 8.12.4612.8838) to perform citation and journal analyses, and VOSviewer (version 1.6.19; Leiden University, Leiden, Netherlands) for mapping the networking analyses of co-authorship of organizations and countries, and co-occurrence of author keywords. The node size and the thickness of the connecting line in the network visualization mapping of country analysis signify the total link strength (TLS) and collaboration strength of intercountry, respectively. Meanwhile, in the co-occurrence analysis of author keywords, the TLS, and the average year of publication of author keywords were symbolized by the size of the node and color schemes, respectively.

## Results

### Global trends in academic publications

**Figure 2:**
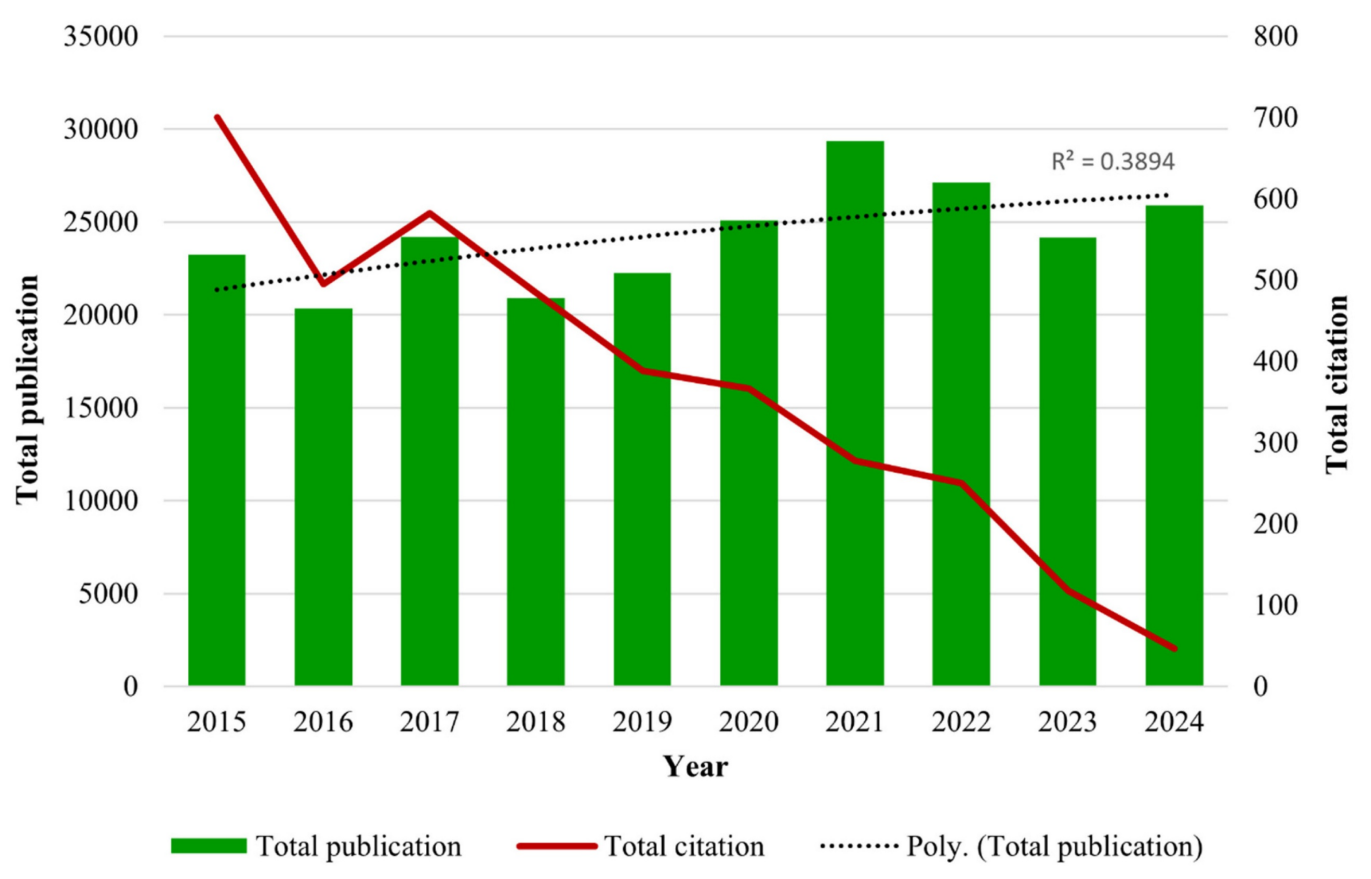
The distribution of annual publications and citations over 10 years.

A total of 7,389 publications were identified from 2015 to 2024 using the search strategy as illustrated in Figure 1. Based on the inclusion and exclusion criteria, 5,544 publications were included in this bibliometric analysis with an average of 29.27 citations per publication. The years 2021 and 2015 documented the highest total publication (TP) (671) and total citation (TC) (30,640), respectively. Over the past 10 years, the fluctuation trends of publication output were observed, following a polynomial pattern with R^2^ = 0.3894. Furthermore, the TC metric showed a consistent declining pattern over the years as well. Total citations showed a decline pattern over the years. There are a few potential factors that are likely contributing to this declining trend, including the citation time-lag effect 31, the shifting of research focus towards treatment management 32, 33, and the Covid-19 impact on scientific publishing 34. The year 2016 not only recorded the lowest TP (465), but it also documented a significant decline of TC within the 10-year period (Fig. 2).

### Prolific organizations and co-authorship analysis

A total of 58 organizations were involved in the research related to genetic variants in acute leukemias. The category of top 10 most prestigious organizations was 60% dominated by the United States with a cumulative TP of 1,324, followed by France (544) and Germany (153), as displayed in Table 1. The University of Texas System, which has nine campuses around the United States, is the most productive organization, with 274 publications, followed by St. Jude Children's Research Hospital (TP = 250) and Institut National de la Santé et de la Recherche Médicale (TP = 228). Interestingly, organizations from the United States also led the top six ranks of the highest TC (Table 1).

Next, these fifty-eight organizations were further analysed for the collaboration between the organizations. Based on this analysis, the United States has consistently become the most influential contributor, as it contributed 60% of the top 20 organizations with a cumulative TLS score of 9,853. The St. Jude Children's Research Hospital, University of Pennsylvania, and University of Texas System are the most superior organizations with the strongest collaboration, as indicated by their TLS values. The University of Pennsylvania, Children's Hospital of Philadelphia, and Pennsylvania Medicine are the most impactful organizations in this field, as they received the highest average citations metric (Table 2). The high average citation reflects the research gaining the most attention based on evaluation of scientific performance.

### Productive journal analysis

Based on the analysis, a total of 5,544 publications that related to genetic variants in acute leukemias were published in 194 different journals. Table 3 listed the top ten of the most productive journals with the highest number of TP. Three publishers from Switzerland, two publishers each from the Netherlands and the United Kingdom, and one publisher each from Italy, Germany, and the United States. The journals from Switzerland including *Cancers, International Journal of Molecular Sciences,* and *Frontiers in Oncology* dominated the category of the most productive journal with a cumulative TP of 434 publications. *Blood* emerges as the most outstanding journal, as it secured the highest metrics of 2024, including journal impact factor (JIF), CiteScore, SCImago journal rank (SJR), and source normalized impact per paper (SNIP). This excellent achievement not only showed that *Blood* has a significant impact on research of genetic variants in acute leukemias, but it is also an influential journal in both the Web of Science and Scopus databases. As displayed in Table 3, the JIF 2024 of these productive journals ranged from 2.2 to 23.1. Six of these journals are ranked in quartile 1. Surprisingly, *Oncotarget* is a journal indexed only in Scopus, and it is also listed in the top ten of most productive journals with seventy-nine publications (Table 3).

### Continents and countries analyses with their global-scale collaborations

**Figure 3:**
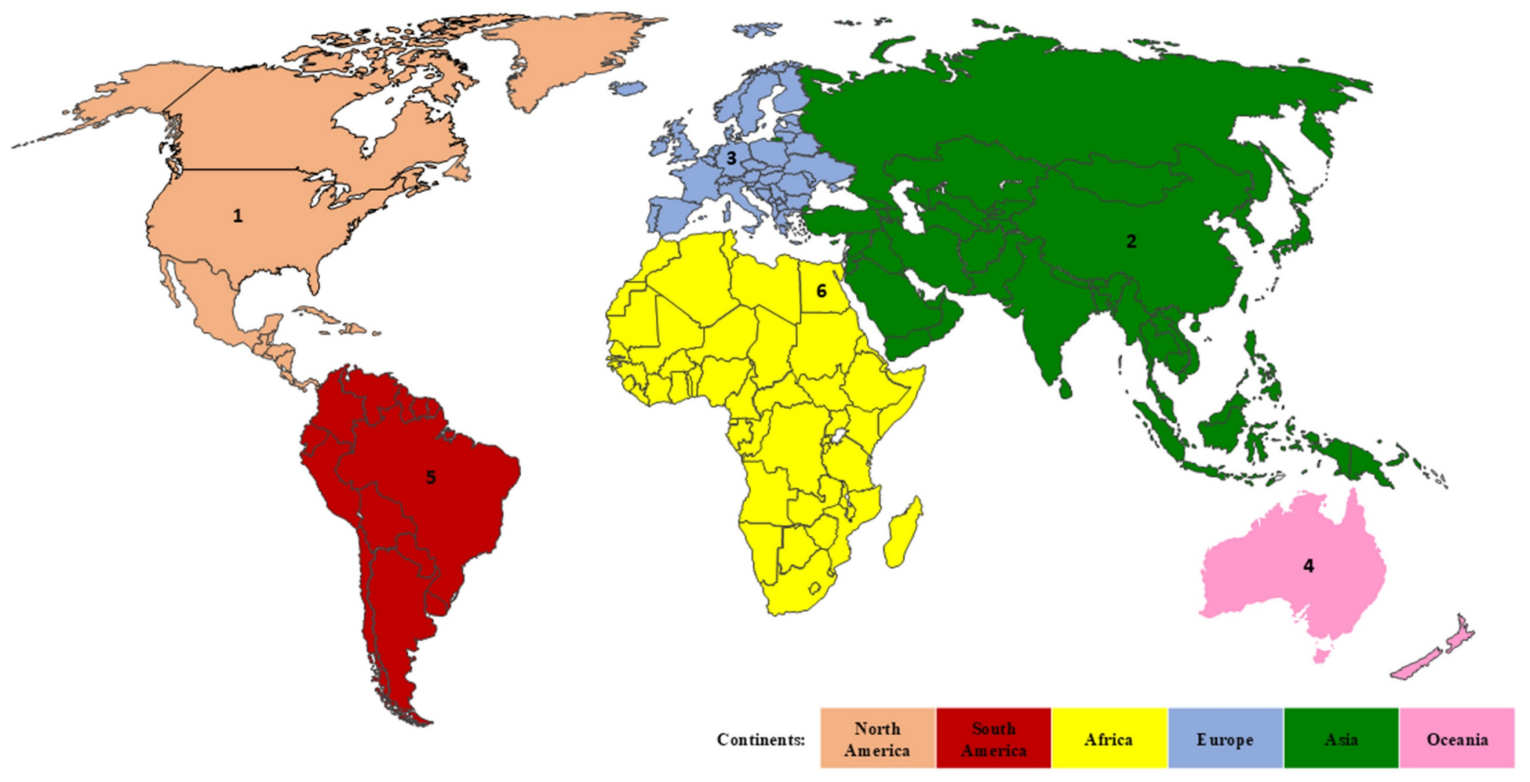
The country productivity according to each continent based on TP metric. Rank 1, 2, 3, 4, 5, and 6 represent the United States, China, Germany, Australia, Brazil, and Egypt, respectively.

Subsequent analysis was conducted aimed at identifying the most productive continent and country in each continent. It was revealed that fifty-five countries from six continents across the globe were productively contributing to the research of genetic variants in acute leukemias. Europe emerges as the most productive and strongest continent with the highest TP (3,629) and TLS (9,371). Figure 3 showed the ranking of the most productive country according to the TP metric in each continent, where the United States is the top contributor, followed by China, Germany, New Zealand, Brazil, and Egypt. The United States consistently maintains its outstanding achievement by gaining the highest values of TLS and citation metrics. Meanwhile, Egypt possesses the lowest values of TP, TC, and citation metrics (Table 4). Furthermore, for a comprehensive analysis across the globe, the United States, Germany, and China are the most productive countries, as they gained the highest TP, TLS, and citations as tabulated in Table 4. On the other hand, Bulgaria (TP: 11; TLS: 19; citation: 95), Tunisia (TP: 19; TLS: 8; citation: 139), and Jordan (TP: 16; TLS: 19; citation: 163) emerged as the underrepresented countries in research, as they have among the lowest values of TP, TLS, and citation metrics.

**Figure 4:**
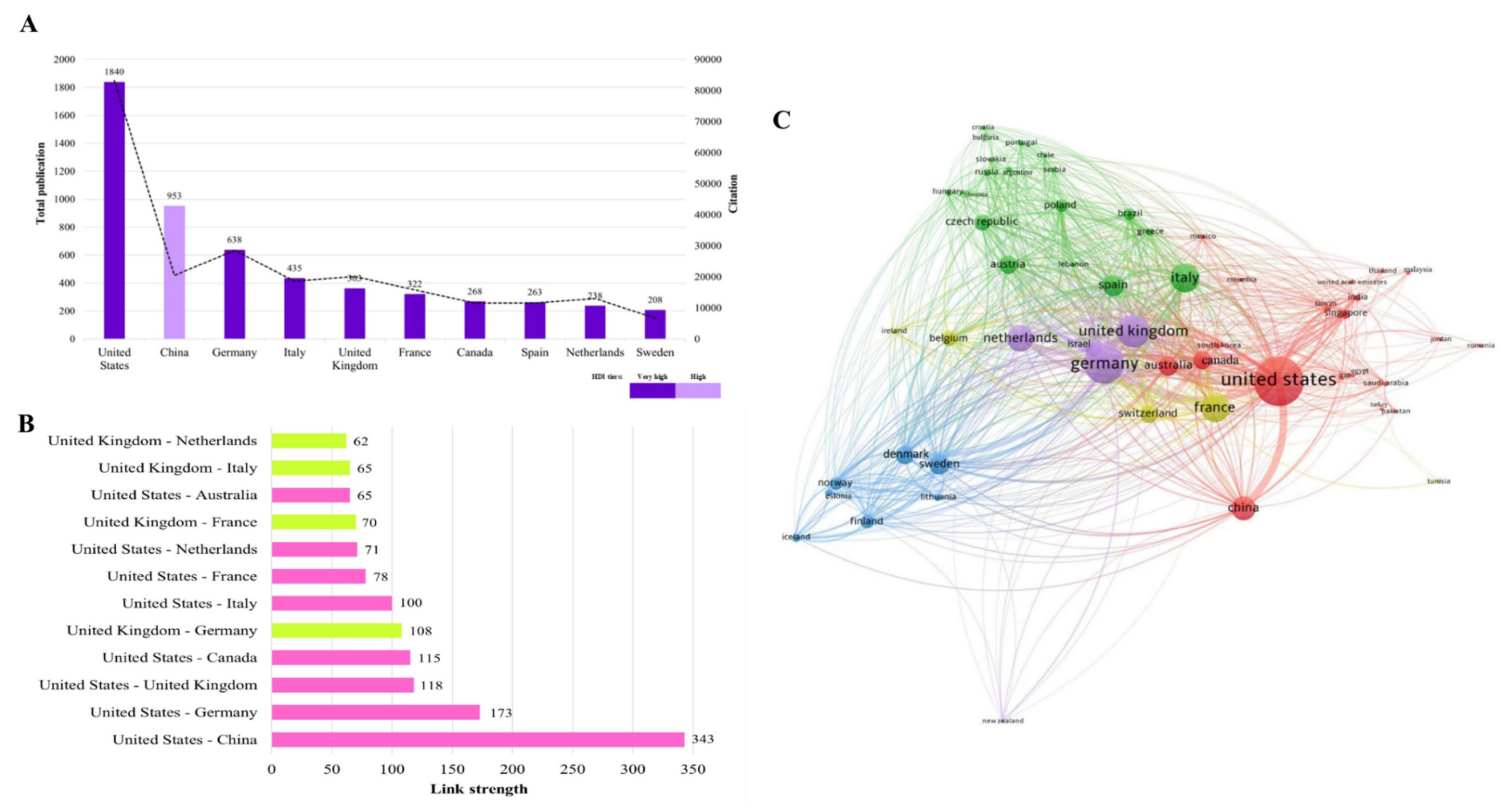
Contributions by countries at a global scale (A) The top 10 most prolific countries (B) The topmost robust collaboration between two countries (C) Network visualization of worldwide intercountry association with a minimum productivity of ten documents. The node size and thickness of the lines signify TLS and the connectivity strength between two countries, respectively.

According to classification by the Human Development Index (HDI), 90% of the top-performing countries were monopolised by the countries that were classified in the very high HDI tier. Intriguingly, China, with an HDI score of 0.797 (high tier), manifests its phenomenal performance by securing the second rank (Fig. 4A). The United States and the United Kingdom are two countries that link to 52 countries (94.5%) across the globe with 1,655 and 949 TLS values, respectively. Figure 4B illustrates that the United States has strong and dynamic international networking with China, Germany, and the United Kingdom as represented by the TLS values. Meanwhile, the TLS value manifested that the United Kingdom develops a vigorous global network with the United States, Germany, and France. As documented, Germany forms an outstanding global collaboration with the United States and the United Kingdom (Fig. 4B). This reflects that Germany is one of the core countries in the global research networks.

Figure 4C visualizes the VOSviewer-generated collaboration map between countries with a minimum productivity of ten documents. The map depicts fifty-five countries classified into five distinct clusters: cluster -1, -2, -3, -4, and -5 are represented by red, green, blue, yellow, and purple colours, respectively. The node size reflects the TLS score, which measures the strength of overall collaboration intercountry. A larger node size indicates a higher extent of global collaboration and vice versa. The United States (TLS = 1,655), Italy (TLS = 801), Sweden (TLS = 483), France (TLS = 788), and Germany (TLS = 1,241) were the countries with robust international collaboration networks from each cluster. The thickness of the lines connecting the nodes represents the link strength of collaboration between two countries. As an example, tangibly, the line between the United States and China (link strength = 343) is the thickest line compared to others. This deciphering the substantial level of research partnerships between these two superpower countries. In contrast, the United States has the lowest link strength value of one with Pakistan and Serbia. It should be acknowledged that the United States has no collaboration with Bulgaria and Tunisia.

### Frequency and co-occurrence analysis of author keywords

**Figure 5:**
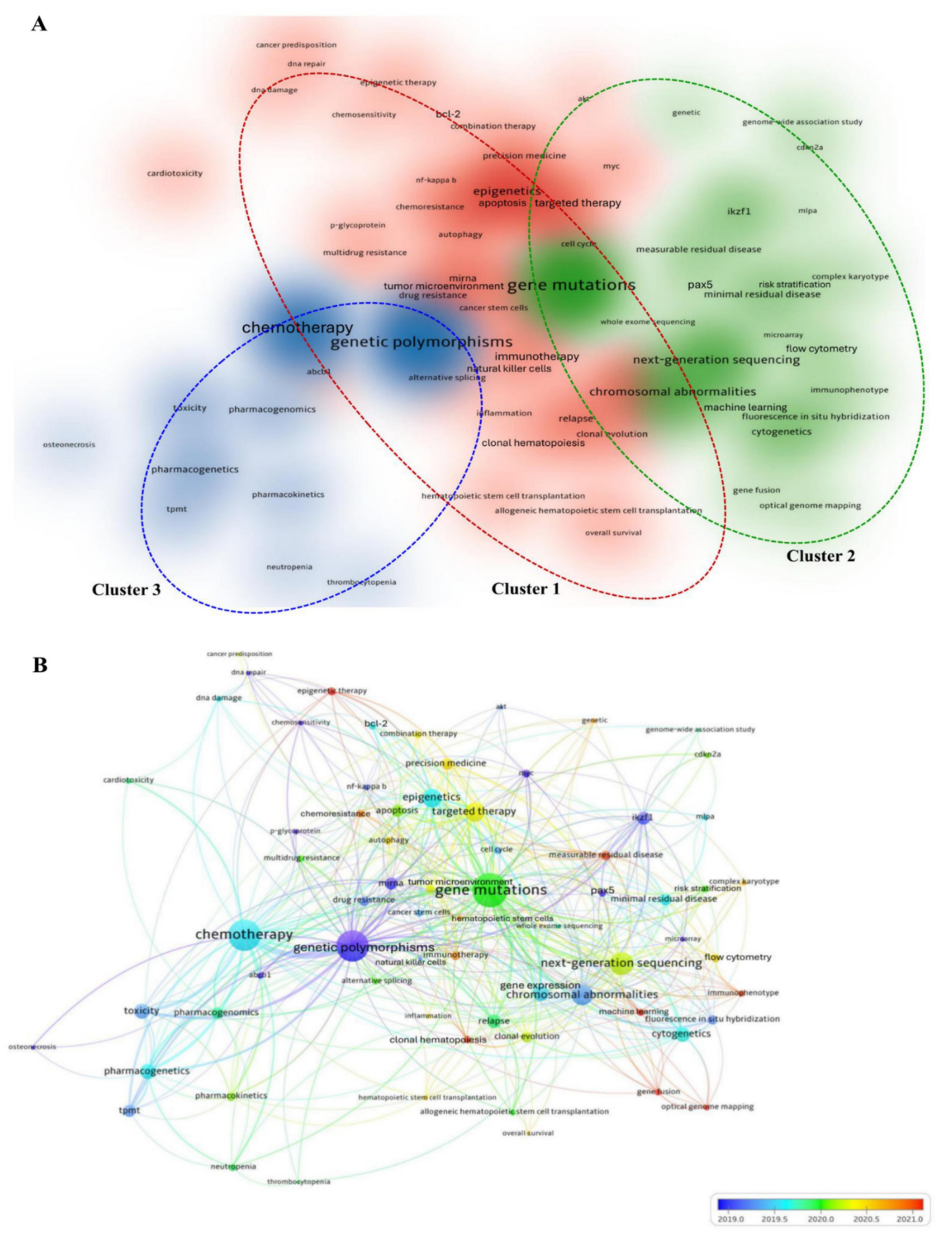
Co-occurrence analysis of author keywords in research related to genetic variants in acute leukemias (A) Cluster density network of most frequent author keywords used research on the topic within 10 years. The colour intensity of each colour correlated with higher co-occurrence frequency. The red, green, blue colors representing Cluster 1 to 3, respectively. (B) Overlay visualization of co-occurrence analysis of author keywords. The size of the node and color schemes represent the value of TLS, and the average publication year of the author keyword (blue: earlier research focus; red: current research focus), respectively.

To determine research progress in genetic variants of acute leukemias from 2015 to 2024, we performed co-occurrence analysis of author keywords using VOSviewer software 35. Sixty-nine keywords out of a total of 7,925 meet the threshold of a minimum number of occurrences of ten per keyword. The unnecessary keywords were removed, and keywords with similar meanings were merged. Figure 5A demonstrated the cluster density visualization of author keywords in which sixty-nine keywords were assigned into three distinct clusters. The closely related keywords were clustered within the same cluster. The density of each color and the distance between the keywords signify the frequency of occurrence of the keyword and the strength between keywords, respectively.

Cluster 1 comprised thirty-seven keywords, and the relevant theme for this cluster was therapeutic strategies and molecular mechanisms in acute leukemias. The most dominant keywords in this cluster were “epigenetics” (173 times), followed by “targeted therapy” (129 times), “gene expression” (87 times), “apoptosis” (80 times), and “miRNA” (66 times). Cluster 2 consisted of twenty-two keywords, related to genomic profiling and risk assessment in acute leukemias. The keyword “gene mutations” is not only the prominent keyword in this cluster, but it is also the most superior in the cluster density network with 452 times of occurrences. Regardless of the numbers of author keywords in each cluster, Cluster 2 is the most significant cluster, as it received the highest values of TLS (1,065) and occurrences (1,263). Cluster 3 contained ten keywords primarily associated with pharmacogenomics and treatment-related toxicity in acute leukemias. In this cluster, “genetic polymorphisms” (429 times) is the most prominent keyword, as illustrated by the most density of blue colour (Fig. 5A).

To determine the research trends and the connectivity between author keywords over time, the overlay map of co-occurrence analysis was generated (Fig. 5B). Based on Figure 5B, the research trends emerged from genetic variations and transcriptional regulation in leukemogenesis (early publications: blue color) to integrated diagnostics and therapeutic strategies in acute leukemias (latest publications: orange color). The overall network analysis revealed that “optical genome mapping,” “machine learning,” and “epigenetic therapy” emerged as the latest research frontiers with the highest score of average publication years (2022.93, 2021.53, and 2021.00, respectively).

Furthermore, the keywords that were classified in the core themes of genetic variants (“gene mutations” (452 occurrences; 2020.00 average publication year), “genetic polymorphisms” (429 occurrences; 2018.98 average publication year), and “chromosomal abnormalities” (161 occurrences; 2019.34 average publication year)), treatment modalities (“chemotherapy” (299 occurrences; 2019.58 average publication year), and “targeted therapy” (129 occurrences; 2020.32 average publication year)), and genomic profiling approaches (“next-generation sequencing” (166 occurrences; 2020.22 average publication year), “cytogenetics” (72 occurrences; 2019.69 average publication year), and “fluorescence *in situ* hybridization” (35 occurrences; 2019.29 average publication year)), and gene regulation mechanisms (“epigenetics” (173 occurrences; 2019.64 average publication year), and “gene expression” (87 occurrences; 2019.52 average publication year)) have the strongest connectivity with other author keywords as reflected by their size of nodes (representing the TLS value).

**Figure 6:**
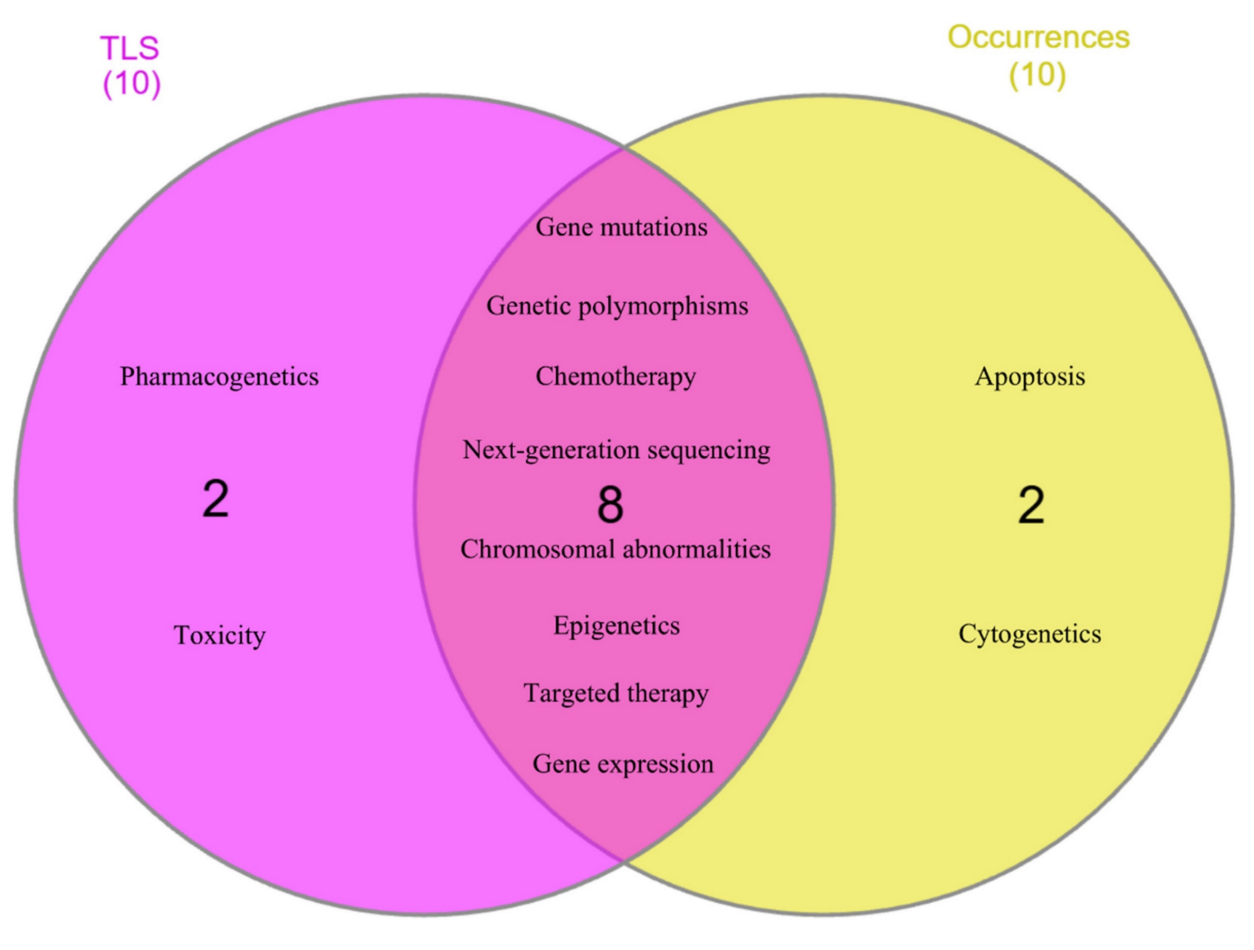
Eight author keywords identified as research hotspots with robust connections.

Based on overall network analysis, “gene mutations,” “chemotherapy,” “genetic polymorphisms,” “next-generation sequencing,” “chromosomal abnormalities,” “targeted therapy,” “epigenetics,” “gene expression,” “pharmacogenetics,” and “toxicity” were identified as the keywords that are highly connected to the research topic, as they had more than 70 of TLS value. Meanwhile, “gene mutations,” “genetic polymorphisms,” “chemotherapy,” “epigenetics,” “next-generation sequencing,” “chromosomal abnormalities,” “targeted therapy,” “gene expression,” “apoptosis,” and “cytogenetics” were determined as core research focus loci, as they had occurrences more than 70 times. Subsequently, these keywords underwent further analysis to determine research hotspots with robust connectivity to the research topic. Figure 6 showed eight keywords, including “gene mutations,” “genetic polymorphisms,” “chemotherapy,” “next-generation sequencing,” “chromosomal abnormalities,” “epigenetics,” “targeted therapy,” and “gene expression,” that achieved the aim of analysis. These keywords manifest remarkable enthusiasm of scholars across the globe between the years of 2015 and 2024.

## Discussion

### Overview of annual publication output

The global trends of publications on genetic variants in acute leukemias from 2015 to 2024 have shown a fluctuating trend, where the years of 2016 and 2021 denoted the lowest and highest total publications. There are few possible justifications year 2016 collectively has the lowest TP including; i) a plethora of significant genetic discoveries that are related to genetic polymorphisms, including key polymorphisms (*NPM1, FLT3, DNMT3A*) in AML and ALL, which have been extensively published in 2015 compared with 2016. This has led to a citation clustering effect in 2015 and overshadowed the year of 2016; ii) the studies conducted prior to 2017, they applied the European LeukemiaNet 2010 (ELN2010) for the diagnosis and guidelines treatments, in which ELN2010 has limited cytogenetic and molecular nuance compared to ELN2017; iii) possibly the year of 2016 was a translational year in which the research emphasis was on the aspect of translation and integration of risk stratification systems and ongoing validation of it in various populations. Despite the year 2016 having the lowest TP, but a paper published by Papaemmanuil and colleagues titled “*Genomic Classification and Prognosis in Acute Myeloid Leukemia*” has significantly impacted the AML landscape 36. The year 2021 possesses the highest total publication, possibly impacted by the updated risk stratification system of ELN2017 37 and the WHO Classification of Tumours of Haematopoietic and Lymphoid Tissues (revised 4^th^ edition, 2017) 38. Both guidelines had revised several significant features and incorporated more cytogenetics and molecular abnormalities into risk stratification systems.

### Analysis of organizations output and their networking

The University of Texas System emerged as a frontrunner of the most productive organization that conducts research on genetic variants in acute leukemias. This university is not only comprised of nine campuses, but it also possesses five health institutions (UT Southwestern, UTMB Galveston, UTHealth Houston, and UT MD Anderson Cancer Center) that are robustly conducting research focused on health and medical sciences. Factors such as the availability of sophisticated technologies, enormous research allocations, and numerous experts across disciplines are among the successful factors towards the productivity of an organization.

Moreover, the United States is not only conquering the most productive organizations, but it is also dominating the most influential organizations globally. As an example, the St. Jude Children's Research Hospital became the most influential organization with the strongest networking, surpassing other world-class organizations such as Inserm and the Md Anderson Cancer Center. There are several potential factors for this robust strength, including research at the St. Jude Children's Research Hospital exclusively focused on hematology-oncology settings. Moreover, this organization had collaborative research with Washington University for an impactful project named the Pediatric Cancer Genome Project. St. Jude collaborates with the World Health Organization (WHO) and global partners to aim to improve survival rates, particularly for pediatric patients worldwide. It also extensively collaborates with institutions in low- and middle-income countries, not only focusing on improving survival rates but also on the quality of care for children that suffer from cancers and other diseases. St. Jude has run several of the largest ALL clinical trials worldwide as an example of total therapy protocols 39, 40.

### Journal analysis

*Blood,* a journal from the Netherlands, became the most productive journal out of 194 journals across the globe that published the publications related to genetic variants in acute leukemias. There are few factors that possibly influence the productivity of a journal including the broad scope of the journal, affordable article processing charge, metrics (quartile, impact factor, CiteScore, acceptance rate), and the effective process of peer review. *Blood* is possibly the core journal for the hematology setting, as reflected by its superior achievement. Intriguingly, despite *Oncotarget* being indexed only by Scopus, it is capable of being listed in the top ten of the most productive journals as well. The possible factor for this might be due to its publication frequency of fifty-two issues per year, the same as the frequency of *Blood*.

### Geographical contributions

Europe has emerged as the most productive and authoritative continent that focuses on research of genetic variants in acute leukemias. The key factors that contribute to the superior achievement of Europe relative to other continents are an impactful European-funded project named BLUEPRINT 41, 42, the establishment of ELN recommendations 37, 43-45, and the establishment of measurable residual disease assessment 46 by European experts, which has contributed on an enormous scale to the acute leukemias setting. Other factors including robust regional and international research collaborations, a large number of research-active countries, and a huge funding capacity across multiple countries also possibly promote this outstanding performance.

Meanwhile, the United States has become the most outstanding country with the highest TP, TLS, and citation values. The primary factors contributing to the exceptional accomplishments of the United States include the successful mapping of the entire human genome through the Human Genome Project 47 and the completed research on thirty-three different cancers, including ten rare cancers in the Cancer Genome Atlas (TCGA) project 48, 49. Undoubtedly, the United States and China are not only among the countries with superpowers, but they also have the most robust dynamic networking, as indicated by their stronger link strength than other nations. Enormous research and development funding budgets, large numbers of research centres and universities, national policies prioritising research output, involvement in global initiatives and multicentre clinical trials, and strong international collaboration policies are the possible factors that contribute to the extraordinary achievement by the United States.

### Author keywords analysis

Co-occurrence analysis of author keywords was performed aimed at discovering the research frontiers and hotspots of genetic variants of acute leukemias in the past 10 years. Out of 7,925 author keywords, 69 keywords met the criteria, and three clusters were generated as the output of the cluster density network. Overlay network uncovered that the early research frontier was concentrated on the discovery phase, where research on identified biomarkers such as genetic polymorphisms, *MYC*, and *ABCB1* were extensively conducted. Meanwhile, the current research focus has emerged on the translational and clinical implementation, including optical genome mapping, machine learning, epigenetic therapy, and immunophenotype. Furthermore, based on the cluster density- and overlay-network, eight keywords, including gene mutations, genetic polymorphisms, chemotherapy, next-generation sequencing, chromosomal abnormalities, epigenetics, targeted therapy, and gene expression, were identified as research hotspots. Three research hotspots with the strongest and most significant connections are further discussed below.

### Gene mutations

By leveraging the current cutting-edge technology, next-generation sequencing (NGS) is competent in determining the most common gene mutations, including *FLT3, NPM1, IDH1/2, DNMT3A,* and *TP53,* in the majority of AML patients. The patient's age and AML status are associated with therapy influencing the frequencies of gene mutations 50. These gene mutations are more prominent manifesting concurrently with other mutations rather than as single mutations 5. In ALL, the most commonly reported gene mutations are *IKZF1, CDKN2A/B*, and *PAX5*. The percentage of *IKZF1* mutations reported ranges from 3.7% to 63%, utilizing various methods including single nucleotide polymorphism (SNP) array 51, real-time quantitative polymerase chain reaction 52, and targeted RNA sequencing 52, 53. Besides, the percentage of *CDKN2A/B* mutation was reported to range from 20% to 39%, and technologies such as fluorescence in situ hybridization (FISH) 54, 55, multiplex ligation-dependent probe amplification (MLPA) 56, and NGS 57 were employed. The gene mutation rate of *PAX5* has been found to range from 31% to 38%, using specific methods including SNP array 58, MLPA 56, FISH, and the integration of quantitative polymerase chain reaction, and target sequencing 59. Not only do selected clinical features act as prognostic factors in risk stratification of AML and ALL, but genetic abnormalities, particularly gene mutations, also contribute as vital indicators 44, 60. Risk stratification is a crucial classification, as it affects the prognostic and treatment decisions. As the example shows, for patients in favorable- and adverse-risk groups, toxicity and relapse are the main obstacles, respectively. According to the ELN 2022 risk stratification guidelines, patients with genetic abnormalities, including t(8;21)(q22;q22) translocation, *NPM1* mutations in the absence of *FLT3*-internal tandem duplication, and bZIP in-frame *CEBPA* mutation, are classified in the favorable risk group. Meanwhile, patients that present genetic abnormalities such as t(6;9)(p23.3;q34.10) translocation, inv(3)(q21.3q26.2), and *TP53* mutation are categorized in the adverse risk group 44. Future research should prioritise this concern, and the identification of novel prognostic factors should significantly enhance the underlying biology of disease, elucidate mechanisms of action of therapeutic agents, and improve the accuracy of prognostic prediction, thereby guiding risk-adapted treatment strategies.

The clinical management of AML followed the ELN risk stratification system. The initial version of the ELN guideline was published in 2010 43. ELN 2022 is the latest and most updated treatment guideline for AML patients, as it revised the previous ELN genetic risk classification, response criteria, and treatment modality suggestions. Noticeably, a significant number of genetic abnormalities are added into this current guideline, and the allelic ratio is removed from the list of ELN 2017 37. This revision reflects the leverage of current sophisticated technologies and advanced understanding of AML. Meanwhile, for ALL clinical management, different regions across the globe follow different guidelines of risk stratification that are designed by several organizations, including the National Comprehensive Cancer Network (NCCN) 40, the European Society for Medical Oncology (ESMO) 61, Berlin-Frankfurt-Münster (BFM) 62, ELN 63, and the United Kingdom National Randomised Trial for Children and Young Adults with Acute Lymphoblastic Leukemia and Lymphoma (UKALL) 64. Several factors, including population-specific data, regulatory and healthcare system structure, and availability of diagnostic resources, have influenced the implementation of specific risk stratification guidelines in different countries. For example, Malaysian hospitals adopted UKALL guidelines in their ALL-clinical management 65, 66. Table 1S (Supplementary Material) provides a summary of the most common gene mutations reported in acute leukemias and also their clinical impacts. Meanwhile, Table 2S (Supplementary Material) showed the overview of clinical trials that were performed for each gene mutation of acute leukemias.

### Genetic polymorphisms

Copy number variations (CNVs; also known as copy number alterations) and single nucleotide polymorphisms (SNPs) are the types of frequent genetic polymorphisms that contribute to acute leukemia. CNVs are not only identified in healthy people who are associated with neuropsychiatric disorders, but they are also currently recognised as a key contributor in several solid tumours and leukemias 67. CNVs occurred in approximately 65% of B-ALL cases 68. CNVs are reported to be implicated in the pathogenesis of diseases, distinct complex-trait diseases 69, disease progression 70, disease prognosis, and therapeutic response 71. CNVs' characteristics are distinct among AML subtypes based on karyotypes, age, and mutations. For example, AML patients with normal karyotypes and t(8;21) and inv(16) showed fewer CNVs, whereas patients with complex karyotypes exhibited more CNVs 72. Unlike AML, CNVs in ALL are more frequently linked to gains or deletions in genes related to cell cycle control and B-cell development. The most often impacted genes are *PAX5, IKZF1, EBF1, CDKN2A/B*, and *RB1*. These CNVs are especially prevalent in B-ALL, where copy number changes in genes related to early B-cell differentiation or cell cycle regulation are present in around 65% of pediatric cases 73. For instance, notably in Philadelphia chromosome-positive (Ph+) or Ph-like ALL subtypes, deletions in *IKZF1*, a gene encoding the transcription factor Ikaros, are closely linked to increased relapse risk, treatment resistance, and worse overall survival.

Furthermore, SNP-based genetic diversity is present in both ALL and AML; the biological pathways impacted by these polymorphisms are different in the two conditions. SNPs are mostly found in genes linked to immune modulation and telomere maintenance in AML, including *CIITA*, *CD200*, *CD163*, *LILRB4*, and *TERT* 74-77. These variations primarily affect treatment results, hematological parameters, and disease susceptibility. As an example, the rs2272022 polymorphism in CD200 has also been associated with variations in platelet levels, suggesting that immune-related SNPs may influence both disease biology and haematological parameters in AML 74. On the other hand, SNPs linked to ALL typically involve genes like *TLR3*, *CDKN2A*, *ARID5B*, and *TERT* that are involved in immunological signalling, lymphoid development, and cell cycle regulation 29, 78-81. These polymorphisms are more closely associated with genetic susceptibility and illness risk; depending on the allele present, certain variations may have protective or risk-enhancing effects. For instance, polymorphisms in the *CDKN2A*, including rs3731217 and rs3731249, have been examined in Caucasian populations. The rs3731217 SNP has been associated with a reduced risk of ALL, suggesting a protective effect, whereas rs3731249 is linked to a significantly increased risk of ALL, with an odds ratio of approximately 2.26, indicating that individuals carrying this variant are more likely to develop the disease 81. Given that the two acute leukemia subtypes have different biological origins and pathogenic mechanisms, AML-related SNPs mainly affect immune-microenvironment interactions and telomere regulation, whereas ALL-related SNPs primarily affect lymphoid differentiation pathways and immune receptor signalling. Table 3S (Supplementary Material) provides further information on recent findings of clinical significance of CNVs and SNPs in the acute leukemia landscape.

### Chemotherapy

Chemotherapy is the standard care for acute leukemias, which consist of three phases of treatment, including induction, consolidation, and maintenance. In AML, the standard backbone of induction therapy is known as the 7 + 3 regimen, which comprises cytarabine and daunorubicin/idarubicin 82. Meanwhile, in ALL, the standard regime used as a chemotherapy backbone consists of vincristine, corticosteroids (dexamethasone/prednisone), and anthracyclines, with or without some form of L-asparaginase 83, 84. Several regimens are widely used across the globe due to distinct subtypes of ALL, and different protocols are proposed by different research groups, such as the MD Anderson Cancer Center, BFM, and Children's Oncology Group (COG) groups. Acute leukemias are potentially curable diseases, as indicated by cure and overall survival rates in younger adult (in AML) and pediatric patients (in ALL); however, these rates are still low in older adult (in AML) and elderly patients (in ALL) 83, 85, 86. Furthermore, relapse, chemoresistance, and infection are the major factors of treatment failure and mortality in acute leukemias 87-90. It is imperative to determine the treatments that are not only effective but also safe for all folks of patient ages across the subtypes of acute leukemias. It is crucial to identify the treatments (single- and multi-agent) that can reduce the risk of relapse, and adverse effects and attain a complete remission. Table 4S (Supplementary Material) provides an overview of current research on discovering the treatments (single- and multi-agent) that are more effective and safer to be incorporated into acute leukemias management.

### Limitations and strengths

Indeed, this study performed a comprehensive analysis, but it is vital to highlight a few limitations so that it could improve in future research. Unquestionable are its advantages; the selection of the English language as the only language in the inclusion criteria might overlook impactful publications in other languages such as Chinese, Spanish, and German. Secondly, this study utilized specific keywords in the search string for both databases, in which the relevant publications that employ different terminologies might have been missed. Lastly, despite data cleaning and harmonization having been performed, some organizations might be spelled differently and also register more than one name into databases. Even though a few constraints were noticed in this study, they did not affect the valuable insights of this research. Firstly, to the best of our knowledge, this is the first bibliometric analysis focusing on the genetic variants in both types of acute leukemias. It is crucial to provide perspective on genetic variants of both types of acute leukemias so that future studies could concentrate on and improve the limitations that are present in both settings of acute leukemias that are restricted towards the era of precision medicine. Besides, this study employed two databases as the sources of data extraction and collection. As for now, there is no available database that is developed for bibliometric analysis. Thus, we utilized two established, user-friendly, and comprehensive databases that aim to maximize coverage, mitigate bias, and minimize missing relevant publications since each of these databases has its own constraints.

Even though the search string incorporated telomerase and its related genes as keywords, but the outputs of the co-occurrence analysis of author keywords did not mandatorily include those keywords. The inclusion of a keyword in the search strategy does not guarantee that it will appear in the co-occurrence network of author keywords analysis. There were several reasons telomerase-related keywords were not visualised in the co-occurrence network analysis of author keywords; (i) Telomerase and its related genes are not part of driver mutations in established classification and risk stratification systems such as WHO, International Consensus Classification, European LeukemiaNet, and National Comprehensive Cancer Network. Consequently, the research intensity focusing on these keywords is not intense compared to driver mutations such as *FLT3*, *NPM1*, and *TP53*; (ii) The frequency of telomerase-related keywords did not meet the minimum occurrence threshold, as this study set the minimum frequency of a keyword at 10; (iii) Research on those telomerase-related keywords was published in a small number of publications; as a result, they are not forming a visible cluster in the co-occurrence network analysis; (iv) The map of the co-occurrence analysis of author keywords tends to reflect the research hotspots and emerging topics as well. Therefore, this study unveils a gap in the research of telomerase gene variants in the acute leukemia landscape, which remains significantly underexplored compared to canonical leukemia driver mutations.

## Conclusions

In a nutshell, this comprehensive bibliometric analysis has effectively manifested research trends and mapped the knowledge structure in the acute leukemia landscape. Undoubtedly, the United States consistently became the most influential and productive country that is focusing on the research core of genetic variants in acute leukemias. Its organizations including the University of Texas System, St. Jude Children's Research Hospital, and the University of Pennsylvania, are among the most prolific organizations that are contributing the massive impacts, particularly for this research focus. The journals that have relevant scope, particularly hematology and covering from basic to clinical studies, are the top sources of genetic variants in acute leukemias research. The findings of this study uncovered author keywords such as gene mutations, genetic polymorphisms, chemotherapy, NGS, chromosomal abnormalities, epigenetics, targeted therapy, and gene expression as the research hotspots. This study also revealed optical genome mapping, machine learning, epigenetic therapy, and immunophenotype, have emerged as research frontiers. These identified research hotspots and frontiers from the literature are representing a subset of the broader mutational panorama reported in genomic studies of acute leukemias. In particular, this study unveils a significant research gap that concentrated on the telomerase-associated genetic variants in the acute leukemia landscape. Thus, it is crucial for future studies to explore this area exhaustively, as it may provide further clarification on the role of telomerase-associated genetic variants within the broader mutational panorama of acute leukemias.

## Supplementary Material

Supplementary tables.

## Figures and Tables

**Table 1:** Top 10 productive organizations in research of genetic variants in acute leukemias.

Organization	Country	TP	*n* (%)	TC
University of Texas System	United States	274	4.94	17317
St Jude Children's Research Hospital	United States	250	4.51	19283
Institut National de la Santé et de la Recherche Médicale (Inserm)	France	228	4.11	9667
Harvard University	United States	224	4.04	18725
Md Anderson Cancer Center	United States	207	3.73	14004
University of California System	United States	196	3.54	14534
University of Pennsylvania	United States	173	3.12	16423
Université Paris Cité	France	159	2.87	6331
Assistance Publique - Hôpitaux de Paris	France	157	2.83	6626
Helmholtz Association	Germany	153	2.76	8348

**Table 2:** Leading organizations in research on genetic variants in acute leukemias.

Organization	Country	TLS	Documents	Average citations
St. Jude Children's Research Hospital	United States	1,065	250	77.13
University of Pennsylvania	United States	1,058	173	94.93
University of Texas System	United States	1,006	274	63.20
University of California System	United States	899	196	74.15
Children's Hospital of Philadelphia	United States	845	121	90.94
Pennsylvania Medicine	United States	845	121	90.94
Harvard University	United States	774	224	83.59
Institut National de la Santé et de la Recherche Médicale (Inserm)	France	747	228	42.40
Md Anderson Cancer Center	United States	734	207	67.65
Université Paris Cité	France	726	159	39.82
Assistance Publique - Hôpitaux de Paris	France	707	157	42.20
University System of Ohio	United States	700	127	53.96
University of California San Francisco	United States	693	114	74.39
Ohio State University	United States	644	96	65.26
Dana-Farber Cancer Institute	United States	590	146	62.49
Helmholtz Association	Germany	586	153	54.56
German Cancer Research Center (DKFZ)	Germany	570	127	60.10
Free University of Berlin	Germany	563	88	57.28
Berlin Institute of Health	Germany	559	87	57.83
Charité - Universitätsmedizin Berlin	Germany	559	87	57.83

**Table 3:** Topmost journals published research related to genetic variants in acute leukemias.

Journal	TP	Country	JIF Quartile^a^	JIF^a^	CiteScore^b^	SJR^c^	SNIP^d^
Blood	180	Netherlands	Q1	23.1	23.0	5.823	3.467
Cancers	174	Switzerland	Q2	4.4	8.8	1.462	1.114
Leukemia	148	United Kingdom	Q1	13.4	18.5	3.458	2.466
International Journal of Molecular Sciences	138	Switzerland	Q1	4.9	9.0	1.273	1.177
Blood Advances	126	Netherlands	Q1	7.1	11.7	2.901	1.659
Frontiers In Oncology	122	Switzerland	Q2	3.3	6.9	1.075	0.860
Haematologica	108	Italy	Q1	7.9	11.3	2.449	1.503
Scientific Reports	102	Germany	Q1	3.9	6.7	0.874	1.213
Leukemia & Lymphoma	92	United Kingdom	Q3	2.2	4.1	0.778	0.604
Oncotarget	79	United States	-	-	5.6	0.785	0.590

^a^Journal impact factor (JIF) data was retrieved from the Journal Citations Report (JCR) 2024; ^b^CiteScore 2024; ^c^SCImago Journal Rank 2024; ^d^Source Normalized Impact per Paper 2024

**Table 4:** The detailed metrics of continents and countries analyses.

Countries	TP	TLS	The most prolific country in each continent			
			Country	TP	TLS	Citation
North America	2,189	2,174	United States	1,840	1,655	83,210
South America	211	428	Brazil	146	242	6,988
Africa	92	45	Egypt	73	37	625
Europe	3,629	9,371	Germany	638	1,241	28,544
Asia	1,879	1,891	China	953	634	20,519
Oceania	195	493	New Zealand	172	459	10,942

## Data Availability

The data that support the findings of this study are available from the corresponding author, Sarina Sulong, upon reasonable request.
