# Supplementary material for: Unraveling Research Trends and Hotspots of Genetic Variants in Acute Leukemias: A Web of Science and Scopus-Based Bibliometric Study
Source: Int J Med Sci. 2026 Mar 30;23(5):1782–97. doi: 10.7150/ijms.128446 (PMC13133873; doi:10.7150/ijms.128446)
Supplement: Supplementary file 1 — Supplementary tables. [file ijmsv23p1782s1.pdf]

Unraveling Research Trends and Hotspots of Genetic Variants in Acute Leukemias: A Web of Science and Scopus-Based Bibliometric Study

Authors

Ninie Nadia Zulkipli, Nur Shafawati Ab Rajab, Nurul Aain Ahmad Fauzi, Amin Abdurrahman Abdul Rashid, Sarina Sulong*

Affiliation

Human Genome Centre, School of Medical Sciences, Universiti Sains Malaysia, Kelantan, Malaysia

Correspondence

Assoc. Prof. Dr. Sarina Sulong

Email: ssarina@usm.my

Supplementary Materials

Table 1S. The summary of the common gene mutations reported in AML and ALL patients.

AML				
Mutation	Functional class	Frequency (%)	Prognostic impact/risks	Description
Class I mutations				
FLT3	Signalling and kinase pathway			The *FLT3* mutation has been determined as a crucial contributor to leukemogenesis associated with AML pathogenesis [1].
FLT3-ITD		20-32 [2, 3]	Unfavourable [1]	*FLT3*-ITD mutation commonly demonstrated adverse clinical features, including increased risk of relapse and leukocyte counts [1].
FLT3-TKD		5-12 [2, 3]	Controversial [1]	*FLT3*-ITD is prominently related to more severe phenotypes [1].<br>AML patients with the *FLT3*-ITD mutation have a high risk of relapse [4] and a low cure rate [5].
RAS	Signalling and kinase pathway	15-47 [6]		*NRAS* mutations are the most prominent RAS mutations in AML patients compared to *KRAS*, and *HRAS* mutations [7].
NRAS		10-15 [2]	Controversial [8]	*HRAS* mutation is infrequent in AML patients [7].
KRAS		5 [7]		*NRAS* and *KRAS* mutations were shown to be insignificantly affecting the outputs of most research that used adult and paediatric cohorts [9].
PTPN11	Signalling and kinase pathway	4-6 [10]	Unfavourable [11]	*PTPN11* mutation is strongly associated with *NPM1* mutation, normal karyotype, older age, CD14 expression, and FB M4/M5 subtypes [10].<br>*PTPN11* mutation is inversely linked with *FLT3*-ITD mutation [10].<br>AML patients with *PTPN11* mutation have distinct molecular and clinical characteristics [11].

Class II mutations				
NPM1	Nucleophosmin	30 [2]	Favourable [12]	One of the most frequent somatic aberrations in AML, particularly in AML patients with normal cytogenetics [10].<br>*NPM1* mutation commonly co-occurs with *FLT3* mutations, specifically the ITD-mutation type [10].<br>*NPM1* mutations are frequently present in adult AML patients of all ages and less common in children, particularly those under three years of age [10].<br>The most prominent of *NPM1* mutations are 4-base pair insertions. These 4-base pair insertions caused the deletion of W288 and W290 (or W290 alone) and the generation of a new C-terminal NES due to the frameshift in the last few C-terminal amino acids [12].
RUNX1	Transcription factor		Unfavourable [13]	*RUNX1* acts as a key contributor to haematopoiesis due to its involvement in the regulation of multiple hematopoietic processes [14].<br>*RUNX1* mutation in AML is linked with unique inferior output and clinicopathologic characteristics [15].
Class III mutations				
DNMT3A	Epigenetic modifier	20 [2]	Unfavourable [16]	The *DNMT3A* mutation is strongly related to *IDH2, NPM1, PTPN11*, and *FLT3*-ITD mutations, higher WBC and platelet counts, older age, and normal and intermediate-risk cytogenetics [16].<br>The *DNMT3A* mutation is inversely linked to *CEBPA* mutations [16].
IDH	Epigenetic modifier	20 [10]	Controversial [17]	*IDH2* mutations are more prominent than *IDH1* mutations, and co-mutation of these mutations is rarely present in the same patient [17].
IDH1		7-10 [2]		In AML patients, *IDH1* and *IDH2* mutations affect the arginine residues at position 132 or 170 (R132 or R170) and 140 or 172 (R140 or R172), respectively [9].
IDH2		10-20 [2]		In patients of intermediate-risk AML with *NPM1* mutation, the R140 mutation in *IDH2* was correlated with a favourable outcome [18].

TET2	Epigenetic modifier	10-17 [2, 3]	Controversial [19]	It was reported that 13.2% of patients with *TET2* mutation were strongly correlated with intermediate-risk cytogenetics, co-mutation with *ASXL1* and *NPM1*, isolated trisomy 8, older age, elevated WBC, and blast counts [20].<br>The frequency of *TET2* mutations is directly proportional with age [21].<br>Deletions (frameshift and non-frameshift), missense, nonsense, splice site mutations are among the most common types present in *TET2* mutations [22].
Class IV mutations				
TP53	Tumour suppressor	2-20 [2]	Unfavourable [23]	Most of the *TP53* mutations are located in exon 5 to exon 8 [10].<br>The *TP53* mutations more commonly occur in older AML patients and therapy-related (30%) or secondary AML (18%) [24].<br>*TP53* mutations are strongly associated with a complex karyotype and are approximately 80% observed in patients with a monosomal karyotype or loss of 7/7q, 5/5q, or 17/7p [25].
WT1	Transcription factor	10 [3]	Unfavourable [26]	Frameshift and missense mutations are commonly present in AML patients and typically in patients with older age, secondary AML, chemoresistance to mainstay treatment, and shorter OS [13].<br>The frequency of co-mutations of *WT1* and *NPM1* in AML patients is ~ 15% [27].
		ALL		
B-ALL				
Mutation	Functional class	Frequency (%)	Prognostic impact/risks	Description
IKZF1	Tumour suppressor gene and transcriptional factor	~15 of paediatric ALL cases	Poor [29]	*IKZF1* mutation is one of the most prominent gene mutations in B-ALL [30].<br>The focal deletion is the most common type of *IKZF1* mutation occurring in 15% of ALL cases and > 50% in high-risk ALL [31].

		~70% (in BCR-ABL1-positive B-ALL cases);<br>~40% (in BCR-ABL1-like B-ALL cases) [28]		Deletions of *IKZF1* were linked with adverse events, poor outcomes, and elevated risk of relapse [32].<br>The most frequent of *IKZF1* deletions determined in B-ALL patients were whole-gene deletions [33], intragenic deletions of exons 4-7 (resulting in the formation of the IK6 isoform), and intragenic deletions of exons 2-7 (removing the ATG start codon located in exon 2) [31].
PAX5	Tumour suppressor gene	~30% [34]	Varies, depending on the type of mutations [35-37]	*PAX5* mutations were postulated as driver mutations in B-ALL leukemogenesis and involved in susceptibility to B-ALL [38].<br>Rearrangements, amplifications, point mutations, and deletions are frequently affecting the *PAX5* gene [39].<br>Rearrangement is the most common mutation in *PAX5* gene that contributes to ALL. As an example, ETV6-PAX5 and ZNF521-PAX5 are the most common fusion proteins that are reported due to rearrangement of the *PAX5* with the *ETV6* and *ZNF521*, respectively [40-41].
CREBBP	Transcriptional co-activator	18% in relapsed paediatric B-ALL patients<br>1% in patients who did not relapse [42]	Poor [43]	Not only did they commonly become bi-allelic during B-ALL evolution, but *CREBBP* mutations were also involved with the activation of the RAS pathway mutation, and this hypothesizes these mutations might stimulate the oncogenic RAS signalling in ALL [44].<br>*CREBBP* loss-of-function (LOF) mutations are identified as recurrent second-hit mutations in various B-ALL subtypes, and they are linked to adverse characteristics [42].
T-ALL				
Mutation	Functional class	Frequency (%)	Prognostic impact/risks	Description

NOTCH1	Oncogene	60% [45]	Controversial [46, 47]	More than 50% of T-ALL patients have activating *NOTCH1* gain-of-function mutations [48].<br>The mutation of *NOTCH1/FBXW7* was identified in at least 60% of T-ALL adult patients [48].
WT1	Tumour suppressor gene	10% [49]	Controversial [50, 51]	In T-ALL, *WT1* mutations are associated with elevated risk of relapse, and chemoresistance [49].<br>The heterozygous frameshift mutations are the most frequent *WT1* mutations identified in T-ALL patients, and are also linked with *HOXA, TLX1*, and *TLX3* [51].
PHF6	Tumour suppressor gene	16%-38% [52]	Favourable (associated with T-cell lymphoblastic lymphoma [53]	Somatic mutations are found in T-ALL patients [54].<br>Small deletions, missense, frameshift, and nonsense mutations are among the alterations that can be found throughout the *PHF6* [54].<br>This mutation is significantly present only in male patients [55].<br>The mutation rate of *PHF6* in T-ALL ranges between 20 and 30% [56].<br>In male patients, it was reported that PHF6 mutations co-exist with *RUNX1, U2AF1*, and *ASXL1* mutations [57].<br>It was reported that *PHF6* mutations were prominently linked with molecular genetic markers including *SET-NUP214* rearrangements, and *JAK1*, and *NOTCH1* mutations [58].

Table 2S. Overview of clinical trials that were performed for the most common gene mutation in acute leukemias.

AML						
Trial, clinical trial ID, study phase	Regimen(s)	No. of patients	Primary objective	Primary endpoints	Conclusion	Ref
			FLT3			
CALGB 10603 (RATIFY), NCT00651261, Phase 3	Standard chemotherapy (daunorubicin + cytarabine + cytarabine consolidation) + either midostaurin/ placebo	717	To examine the effect of the addition of midostaurin to standard chemotherapy in AML patients with an FLT3 mutation	OS	The addition of midostaurin to standard chemotherapy significantly prolonged overall and event-free survival among patients with AML and an FLT3 mutation.<br>In 2017, the US Food and Drug Administration (FDA) and European Medicines Agency approved the combination of midostaurin in a standard chemotherapy regimen in AML patients with an FLT3 mutation.	[59-62]
QuANTUM-First, NCT02668653, Phase 3	Quizartinib vs. placebo	539	To determine the efficacy of quizartinib versus placebo in newly diagnosed AML patients with FLT3-ITD-positive.	OS	This trial showed the improvement in overall survival of this population, where it was indicated by the increase in relapse-free survival and duration of complete remission, and the reduction in cumulative incidence of relapse and MRD underlies the overall survival benefit and the manageable safety.<br>Thus, this inhibitor is potentially to be used for elderly patients (aged 18–75 years) with FLT3-ITD-positive newly diagnosed AML.	[63]
NCT04140487, Phase 1/2	Azacitidine + venetoclax + gilteritinib	52	To evaluate the efficacy and safety of these combination regimens in AML patients with FLT3 mutation.	MTD (Phase 1)<br>OR (Phase 2)	Appropriate dosage modifications were needed due to myelosuppression in most of the patients.<br>This combination regimen was effective and safe, particularly in the frontline cohort, resulting in a CR rate and OS of 90% and 72%, respectively.	[64]

			NPM1			
QUAZAR AML-001, NCT01757535, Phase 3	Azacitidine	472	To identify the effects of oral AZA vs. placebo in patients with NPM1 mutation at AML diagnosis	OS	Patients with $NPM1^{mut}$ had longer median OS than patients with $NPM1^{wd}$ (47.2 months vs. 19.6 months, respectively).<br>$NPM1^{mut}$ patients had significantly longer median relapse-free survival (RFS) compared to $NPM1^{wt}$ patients in both the Oral-AZA (54% improvement) and placebo (35% RFS improvement) arms.	[65]
NCT00893399, Phase 3	GO + standard chemotherapy (idarubicin, etoposide, cytarabine, ATRA, pegfilgrastim) vs. standard chemotherapy (idarubicin, etoposide, cytarabine, ATRA, pegfilgrastim)	588	To determine the efficacy of combination regimens of GO with standard therapy and ATRA in AML patients with NPM1 mutation within the randomized AMLSG 09-09 trial.	EFS	The trial failed to achieve the primary endpoint of EFS by the addition of GO into combination regimens in AML patients with NPM1 mutation, where the combination resulted in a higher death incidence.	[66]
			IDH			
NCT03173248, Phase 3	Ivosidenib + azacitidine vs. placebo + azacitidine	146	To identify the efficacy of the combination of ivosidenib and azacitidine compared to placebo and azacitidine in newly diagnosed AML patients with IDH1 mutation who are ineligible for intensive induction chemotherapy.	EFS	The combination of ivosidenib-and-azacitidine significantly improved the EFS compared to placebo-and-azacitidine group.	[67]
NCT03683433, Phase 2	Azacitidine + enasidenib *Continuously BCL2 inhibitor (venetoclax) and FLT3 inhibitor	26	To evaluate the efficacy of this combination regimen in AML (newly diagnosed and relapsed/refractory) patients with IDH2 mutation who	CRc	This combination regimen showed a promising effectiveness in AML patients.	[68]

	(sorafenib/gilteritinib/midostaurin) were allowed		ineligible for intensive chemotherapy.			
DNMT3A						
NCT00492401, Phase 2, and NCT00703300, Phase 1	Decitabine vs. decitabine + bortezomib	46	To evaluate DNMT3A mutational status in older, previously untreated patients with AML	CR[a]; mOS	AML patients with DNMT3A mutation have longer CR rates and mOS than patients with DNMT3A wild type (CR rate: 75% vs. 34%; mOS: 15.2 months vs. 11.0 months, respectively).<br>It was postulated that AML patients with DNMT3A mutations—particularly those with R882 mutations and/or co-mutated with NPM1—may show a promising response to decitabine treatment.<br>The limitation of this study was the limited number of AML patients with DNMT3A mutation. Thus, future study is suggested to be performed in a large number of AML patients with DNMT3A mutation.	[69]
TP53						
NCT01773408, Phase 1	Idasa vs. Idasa-C	122<br>*Patients with confirmed AML:r/r AML, sAML, t-AML, and de novo AML	To identify the safety and pharmacokinetics of escalating doses of Idasa as a monotherapy or combination regimens of Idasa-C in AML patients	MTD; RP2D; DLTs; AEs; SAEs	The study showed promising outcomes, and further research on the development of idasanutlin is highly recommended.	[70]
Beat AML Master Trial,	Entospletinib + decitabine	58	To determine the efficacy of entospletinib + decitabine in AML patients	CRc; ORR; mDoR; mOS	A novel treatment modality is urgently needed, as this trial resulted in ineffective outcomes, as	[71]

NCT03013998, Phase 2					indicated by the low and short CR rate and short OS, respectively.	
NCT01515527, Phase 2	Cladribine + LDAC alternating with decitabine	118	To evaluate the efficacy of this regimen in AML patients	CR[b]; DFS	Further confirmatory testing is needed, as this trial demonstrated promising efficacy of this regimen in a cohort of older and/or unfit patients with newly diagnosed AML.	[72]
NCT04214860, Phase 1	Eprenetapopt + azacytidine + venetoclax	49	To assess the safety and preliminary efficacy of this regimen in AML patients	DLTs; TEAEs; SAEs	The findings of this study support further evaluation of this regimen in the treatment of TP53-mutated AML.	[73]
ALL						
B-ALL						
Trial, clinical trial ID, study phase	Regimen(s)	No. of patients	Primary objective	Primary endpoints	Conclusion	Ref
NCT02101853, Phase 3	Reinduction chemotherapy (vincristine, dexamethasone, pegasparagase, mitoxantrone) + blinatumomab/chemotherapy + hematopoietic stem cell transplant (HSCT)	208	To identify the substitution of blinatumomab for intensive chemotherapy in consolidation treatment would improve survival in patients with B-ALL first relapse.	DFS	The B-ALL patients in the blinatumomab group resulted in an insignificant difference in DFS compared with the chemotherapy group. Further clinical trials are suggested to be performed using a larger number of participants.	[74]
NCT00381680, Phase 3	High-/standard vincristine dosing + different combination chemotherapy regimens (prednisone + doxorubicin hydrochloride + pegaspargase +	271	To assess the efficacy of high vincristine dosing compared with standard vincristine dosing in patients with intermediate-risk relapse of B-ALL	EFS; OS	The EFS and OS of the AALL0433 trial (63.6% and 72.3%, respectively) showed a similar output as in the UK ALLR3 trial (60% and 70%, respectively). It was postulated higher vincristine dosing improved the outcomes. However, the ALLR3 trial also demonstrated significant infectious toxicities.	[75]

	cytarabine + methotrexate + dexamethasone + etoposide + cyclophosphamide +leucovorin calcium + filgrastim + asparaginase +mercaptopurine)					
EWALL-BOLD, NCT03480438, Phase 2	Blinatumomab<br>*Patients received standard-of-care chemotherapy before, between, and after blinatumomab cycles.	62	To examine blinatumomab in sequence with chemotherapy in newly diagnosed older B-ALL patients.	CR[c]	This finding revealed that alternating between standard chemotherapy and blinatumomab showed potential efficacy and tolerability with a low mortality and high molecular response and cytologic for this age group.	[76]
T-ALL						
Trial, clinical trial ID, study phase	Regimen(s)	No. of patients	Primary objective	Primary endpoints	Conclusion	Ref
NCT01085617, Interventional phase	SOC +/- nelarabine	144	To evaluate the addition of nelarabine to SOC to improve the efficacy for T-ALL patients.	ERS; OS	The addition of nelarabine into SOC did not show the benefit to EFS or OS.	[77]
NCT00501826, Phase 2	Hyper-CVAD + nelarabine + PegAsP	145	To identify the CR[a] post-treatment with hyper-CVAD in combination with nelarabine and PegAsP in previously untreated patients with T-ALL and T-LBL	CR[a]	The addition of venetoclax to regimens of hyper-CVAD-nelarabine-PegAsp showed promising outcomes for adult patients with T-ALL/LBL.	[78]

NCT00558519, Phase 2	Paediatric regimen: *Different treatment phases have different combination regimens (6-MP, 6-TG, Ara-C, CTX, DEX, DNR, MTX, PegAsP, pred, and VCR)	295	To determine the efficacy and tolerability of using a paediatric regimen for older AYAs with newly-diagnosed ALL	CR[b]; EFS; DFS; OS	This finding showed that the implementation of paediatric regimen was effective and tolerable, as indicated by the improved EFS and OS of this population (up to the age of 40 years old) compared to the historical cohort (Children's Oncology Group randomized study AALL0232).	[79]
NCT02518113, Phase 1	Crenigacestat (50-, 75-, 100-, 125-mg) + DEX	36	To identify the RP2D of crenigacestat in combination with DEX in adult, relapsed/refractory T-ALL/T-LBL patients.	DLTs	This finding established 75 mg 3 times per week as the RP2D of Crenigacestat in combination with DEX for this population.	[80]

6-MP: 6-mercaptopurine; 6-TG: 6-thioguanine; AEs: Adverse events; AML: Acute myeloid leukemia; APL: Acute promyelocytic leukemia; Ara-C: Cytarabine; ATRA: All-trans retinoic acid; AYAs: Adolescents and young adults; CTX: Cyclophosphamide; CR[a]: Complete remission rate; CR[b]: Complete response rate; CR[c]: Complete hematologic remission; CRc: Composite complete remission rate; DEX: Dexamethasone; DFS: Disease-free survival; DLTs: Dose-limiting toxicities; DNR: Daunorubicin; EFS: Event-free survival; GO: Gemtuzumab ozogamicin; hyper-CVAD: Hyperfractionated cyclophosphamide; vincristine sulfate; doxorubicin hydrochloride; and dexamethasone; Idasa: Idasanutlin; Idasa-C: Idasanutlin plus cytarabine; LDAC: Low-dose cytarabine; mDoR: Median duration of response; mOS: Median overall survival; MTD: Maximum-tolerated dose; MTX: Methotrexate; ORR: Overall response rate; OS: Overall survival; PegAsP: Pegylated asparaginase; pred: Prednisone; RP2D: Recommended phase 2 dose; r/r AML: Relapse/refractory AML; sAML: Secondary AML; TEAEs: Treatment-emergent adverse events; t-AML: Treatment-related AML; T-LBL: T-cell lymphoblastic lymphoma; SAEs: Serious adverse events; sAML: Secondary acute myeloid leukemia; SOC: Standard chemotherapy; VCR: Vincristine.

Table 3S. The epitome of the clinical impact of the present CNVs and SNPs that are implicated in acute leukemias across the nations.

CNVs in AML					
CNVs	Population	Objective	Findings	Conclusion	Ref
SEMA4D, CBFB, CHAF1B, SAE1, and *DNMT1*	Mixed (Global)	To identify genes linked to CNVs whose presence or absence (or gain or loss) correlates with overall survival, as well as genes whose expression, influenced by these CNVs, is itself a prognostic factor.	102 CNV-related genes were found whose copy number status (gain/loss) was associated with patient survival.<br>Five genes (SEMA4D, CBFB, CHAF1B, SAE1, and DNMT1) were identified whose expression is modulated by the CNVs and whose expression is significantly associated with clinical outcomes.	The research suggests that analysing CNV profiles, along with examining the expression changes they cause, can offer prognostic indicators in AML. Specifically, the five genes they discovered could act as new prognostic markers.	[81]
ALOX15B, MTDH, DNAJB6, HSPB1, ATF4, and *PLIN2*.	Mixed (Global)	To investigate the prognostic role of ferroptosis-related genes (FRGs) *driven by* CNVs in AML.<br>To build a prognostic model/gene signature that combines CNV data and expression of FRGs that can better predict survival in AML patients.	They identified six CNV-driven ferroptosis-related genes (FRGs). These FRGs, whose expression is altered in AML and correlates with CNV status, include ALOX15B, MTDH, DNAJB6, HSPB1, ATF4, and PLIN2.<br>They refined the prognostic model to focus on two genes: *DNAJB6* and *HSPB1*. *DNAJB6* was identified as a protective factor, with higher expression levels linked to improved survival, while *HSPB1* was associated with poorer survival outcomes, as increased expression indicated a higher risk in their risk-score model.	Their two-gene model (*DNAJB6* and *HSPB1*) based on CNV-driven ferroptosis-related genes offers a *novel signature* with good prognostic power in AML.	[82]
NPM1, followed by *FLT3*, *DNMT3A*, *TET2*	European	To evaluate a rapid, robust, high-throughput protocol for detecting both gene mutations and copy number changes in	The most frequent mutations were *NPM1*, followed by *FLT3*, *DNMT3A*, and *TET2*.	HaloPlex is a quick and reliable target enrichment method that can aid diagnosis and prognostic	[83]

		AML in a diagnostically suitable manner.	*DNMT3A* mutations can persist post-chemotherapy and in two cases studied at diagnosis and relapse.	stratification of acute myeloid leukemia patients.	
CNVs in ALL					
CNVs	Population	Objective	Findings	Conclusion	Ref
PAX5, *IKZF1*, *EBF1, CDKN2A/B*, *RB1*	Mixed	To examine and integrate existing research on the impact of CNVs, particularly gene deletions and gains, on the prognosis of B-ALL.<br>To explore which CNVs are most prevalent in paediatric versus adult B-ALL, how CNV patterns change from diagnosis to relapse, and which CNVs are currently utilized or should be considered for risk stratification.	In paediatric B-ALL, about 65% of cases harbour CNVs in genes related to early B-cell differentiation (e.g., *PAX5*, *IKZF1*, *EBF1*) or cell cycle regulation (e.g., *CDKN2A/B*, *RB1*).<br>Among adult B-ALL, deletions in *IKZF1*, *CDKN2A/B*, *PAX5*, etc., are also frequent, especially in subtypes such as Ph+ or Ph-like B-ALL.<br>IKZF1 deletion: associated with worse outcome (relapse risk, drug resistance, overall survival) in many studies, particularly in podiatric patients and in Ph+ or Ph-like disease.<br>CDKN2A / CDKN2B deletions: These are frequent, and in many studies, particularly in adult Ph− B-ALL, their presence correlates with worse outcomes (lower OS or higher relapse risk).<br>PAX5 deletions: Their prognostic impact seems to depend on whether they co-occur with other adverse CNVs (e.g. *IKZF1*) or in specific subtypes. Alone, *PAX5* deletions sometimes show a weaker prognostic signal.	CNVs (especially deletions in *IKZF1*, *CDKN2A/B*, *PAX5*) are important prognostic markers in B-ALL. They are common and, in many studies, significantly associated with higher relapse risk, worse event-free survival (EFS), and overall survival (OS).	[84]
TERT gene polymorphisms in AML					

Polymorphism	Population	Objective	Findings	Conclusion	Ref
Rs3087456, rs4780335 (in *CIITA*); rs2272022, rs3746444 (in *CD200*); rs4883263 (in *CD163*); rs1048801 (in *LILRB4*)	Chinese	To investigate the involvement of AML immunosuppression-related SNPs on the aetiology and treatment efficacy heterogeneity of AML	This study showed four SNPs involved in the aetiology and treatment efficacy of AML. The details are as below:<br>Rs4883263 in *CD163* correlated with AML susceptibility, abnormal chromosome karyotype, and peripheral blood PLT count.<br>Rs4780335 in *CIITA* is linked to peripheral blood WBC count and AML OS.<br>Rs2272022 in *CD200* is linked to peripheral blood PLT count.<br>Rs1048801 in *LILRB4* is associated with AML OS and AML treatment sensitivity.	The involvement of AML immunosuppression-related SNPs serves as crucial indicators for predicting treatment outcomes in AML patients.	[85]
Q53H, V170M, A184T, S255Y, A288V, H412Y, I540M, R631W (nsSNPs of TERT gene)	Computational analysis	To classify the harmful TERT gene mutations, and to analyse them using various computational approaches at structural, functional, and translational expression levels	Q53H, V170M, A184T, S255Y, A288V, H412Y, and I540M all negatively impacted protein stability and hydrophobicity, protein-protein and protein-nucleic acid interactions, protein folding, three-dimensional structure, secondary structure, and conservation profile.	These SNPs may be employed as possible targets in biological markers, protein research, and illness diagnostics.	[86]
Rs2853669	Multinational	To determine the genetic predisposition to AML, their association with different prognostic markers, and their impact on survival, outcome, and the prognosis of affected patients	According to multivariate Cox regression, rs2853669 was a significant predictor of overall survival in AML patients.<br>The estimated adjusted hazard ratio revealed that survival time changed negatively with the rs2853669 mutation (HR adjusted = 1.54, 95% CI: 1.01-2.35).	The TERT rs2853669 variant genotype had a negative effect on AML patients' overall survival in the presence of other known prognostic factors.	[87]

Rs2853669, rs2736100	Chinese Han	To identify the association between TERT gene polymorphisms and AML susceptibility in a Chinese Han population	Rs2853669:<br>The frequency differences between GG and AA genotypes were not significant.<br>The frequency of G allele showed a decreasing trend in the case group but was not statistically significant.<br>The Rs2853669 polymorphism is located at 245 bp from the TERT ATG site.<br>Rs2736100:<br>The CC genotype was higher in AML patients.<br>Individuals with the CC genotype showed a 2.632-fold higher risk of AML.<br>The frequency of the C allele also higher in the AML case group.<br>The Rs2736100 polymorphism is located in the second intron of the TERT gene.	This study proposed a positive correlation between the susceptibility to AML and the rs2736100 polymorphism of the TERT gene in the Chinese Han population.	[88]

TERT SNPs and their association with ALL

Polymorphism	Population	Objective	Findings	Conclusion	Ref
Rs2735940, rs2736100 and rs10069690	Chinese	To investigate the association of TERT polymorphisms with risk of childhood ALL	For allele comparison, rs2736100, and rs10069690 along with rs2735940, were associated with the risk of developing ALL in children (P=0.036, 0.011, and 0.022, respectively).<br>According to in vitro luciferase assays performed in Jurkat cells, the T allele of rs2735940 had greater transcriptional activity than the C allele.	The TERT promoter rs2735940 polymorphism may affect TERT activity.<br>Rs2736100 may be associated with telomere function, making it a potential biomarker for genetic susceptibility to ALL in Chinese children.	[89]

			The T allele of rs2735940 had greater TERT mRNA expression, as suggested by analyses of the bone marrow.		
Rs2735940; MNS16A Ins/Del	Iranian	To investigate the association of the SNPs -1327C/T and MNS16A Ins/Del, and telomere length with risk of paediatric ALL	There were no associations made concerning hTERT gene variants or haplotypes with the risk of childhood ALL. Also, hTERT polymorphisms did not correlate with RTL or the clinicopathological features of the patients, including age (P=0.304), sex (P=0.061), organomegaly (P=0.212), CSF involvement (P=0.966), or treatment response (P=0.58).	Independent of TERT variations, telomere attrition may be connected to the pathophysiology of paediatric ALL.	[90]

Polymorphisms from other genes involved in AML and ALL

Polymorphism	Population	Objective	Findings	Conclusion	Ref
Rs2071746 (in *HMOX1*); rs9245, rs7211 (in *TXNIP*); rs12488654 (in TNFS10/TRAIL); rs1132339 (in *TNFAIP2*)	Chinese	To investigate the association between SNPs in immunomodulatory factors and AML	Rs2071746 (*HMOX1*) and rs1132339 (*TNFAIP2*) are linked with BM blasts at the time of AML patient diagnosis.<br>Rs7211 (*TXNIP*) is linked with treatment sensitivity caused by cytarabine and anthracyclines in AML, and rs9245 (*TXNIP*) is linked with adverse outcomes associated with AML recurrence.<br>Overall survival of the AML patients is associated with the AA genotype of TRAIL/TNFSF10 rs12488654, which may be an independent favourable factor for AML prognosis	SNPs in the *HMOX1, TXNIP,* TNSF10/TRAIL, and *TNFAIP2* genes are linked to AML and serve as a crucial reference for predicting the prognosis and responsiveness to therapy of AML patients.	[91]
Rs4132601, rs11978267 (in *IKZF1*)	Various ethnicity	To investigate the association between ALL susceptibility and *IKZF1* gene SNPs	A significant association was found between rs4132601 and ALL across genetic models.	The *IKZF1* rs4132601 mutation is a major genetic risk factor correlated to ALL. Diverse study is needed for a full understanding	[92]

				and improved diagnostic techniques, even if these findings support the use of rs4132601 in genetic risk profiles for ALL.	
Rs3775296 C/A, rs5743312 C/T, rs3775291 C/T, and rs3775290 C/T (in *TLR3*)	Saudi	To investigate the association between specific SNPs in the TLR3 gene and susceptibility to ALL in the Saudi population.	There is a strong correlation between a greater risk of ALL and rs5743312 (C/T). Those with the T allele were more likely to develop ALL than controls.<br>Given that it is associated with a decreased incidence of ALL, rs3775290 (C/T) could have a protective effect.	In the Saudi population, there is a substantial correlation between the risk of ALL and certain *TLR3* genetic variations (rs5743312 and rs3775290).<br>The findings highlight the potential of *TLR3* SNPs as biomarkers for ALL susceptibility and encourage further research into their physiological and clinical importance.	[93]
Rs5743618 (in *TLR1*); rs4986790, rs4986791 (in *TLR4*); rs5744105 (in *TLR5*); TLR6: rs5743810 (in *TLR6*); rs5743836, rs187084 (in *TLR9*); rs2569191 (in CD14-159)	Brazilian Amazon	To investigate the association between specific SNPs in the TLRs gene and susceptibility to ALL in the Brazilian Amazon	An increased risk of getting ALL is strongly linked to TLR6 (rs5743810, C>T) (OR: 3.20, 95% CI: 1.11–9.17, p = 0.003). Additionally, in ALL individuals, it is associated with protection against mortality (OR: 0.48, 95% CI: 0.24–0.94, p = 0.031). Another risk factor for ALL is TLR9 (rs187084, C>T) (OR: 2.29, 95% CI: 1.23–4.26, p = 0.000). Protection against mortality in ALL is linked to TLR1 (rs5743618, T>G) (OR: 0.17, 95% CI: 0.04–0.79, p = 0.008).	Although some *TLR1* and *TLR6* mutations may be conferring protective effects against mortality, polymorphisms in *TLR6* and *TLR9* genes are associated with increased risk of ALL. These findings underscore the importance of various immune gene variations for outcomes and susceptibility to ALL, and require future studies in diverse populations.	[94]
Rs7073837, rs10740055, rs7089424, rs10821936, rs4506592, rs10994982, rs7896246, rs10821938, rs7923074, rs6479778, rs4948487, rs6479779,	Yemeni	To examine the association of *ARID5B* SNPs with ALL risk among Yemeni children, providing new insights for the Arab population	Out of 14 *ARID5B* SNPs genotyped, nine (rs7073837, rs10740055, rs7089424, rs10821936, rs4506592, rs10994982, rs7896246, rs10821938, rs7923074) were significantly associated with ALL under additive genetic models.	Many variations of the *ARID5B* gene are significantly associated with ALL risk in children from Yemen, and many of the associated SNPs have gender-specific effects.	[95]

rs2893881, and rs10994990 (*ARID5B* intronic SNPs)			Rs10740055, rs10994982, and rs6479779 were significant in females, while rs10821938 and rs7923074 were significant in males under the recessive model. Under the dominant model, rs7073837, rs10821936, rs7896246, and rs6479778 were significant in males only.<br>The additive model revealed that rs10821936 was significant in both genders.		
Rs3731217, rs3731249 (in *CDKN2A*)	Caucasian	To investigate the association of two key SNPs in *CDKN2A* with ALL susceptibility	Rs3731217: This SNP is associated with a reduced risk of ALL (OR = 0.72), suggesting a protective effect.<br>Rs3731249: This SNP is associated with a significantly increased risk of ALL (OR = 2.26), indicating it is a strong risk factor.	Rs3731217 and rs3731249, are significantly associated with ALL risk, with effects most evident in Caucasian populations.<br>Although further study is needed to validate their impact in other ethnic groups, these variations may be significant genetic indicators for ALL risk.	[96]

ALL: Acute lymphoblastic leukemia; AML: Acute myeloid leukemia; ARID5B: AT-rich interaction domain 5B; B-ALL: B-cell acute lymphoblastic leukemia; CDKN2A: Cyclin dependent kinase inhibitor 2A; CI: Confidence interval; CSF: Cerebrospinal fluid; EFS: Event-free survival; HMOX1: Heme oxygenase 1; HR: Hazard ratio; hTERT: Human telomerase reverse transcriptase; IKZF1: IKAROS family zinc finger 1; nsSNPs: Non-synonymous single nucleotide polymorphisms; OR: Odds ratio; OS: Overall survival; RTL: Relative telomere length; SNPs: Single nucleotide polymorphisms; TLR1: Toll-like receptor 1; TLR3: Toll-like receptor 3; TLR4: Toll-like receptor 4; TLR5: Toll-like receptor 5; TLR6: Toll-like receptor 6; TLR9: Toll-like receptor 9; TLRs: Toll-like receptors; TNFAIP2: Tumour necrosis factor alpha induced protein 2; TNFS10/TRAIL: Tumour necrosis factor superfamily member 10/tumour necrosis factor-related apoptosis-inducing ligand; TXNIP: Thioredoxin interacting protein.

Table 4S. The summarise of current research on the effectiveness and safety of single- and multi-agents in acute leukemias setting.

AML					
Clinical trial ID, study type, phase	Chemotherapeutic agent(s)	Objective	Primary endpoint	Conclusion	Ref
NCT02416388, Interventional Phase II/III	IDAC vs. HDAC	To compare IDAC with HDAC as postinduction therapy in patients 18 to 60 years of age with ND-AML.	OS	In this randomized clinical trial, OS at 5 years was noninferior for intermediate-dose Ara-C compared with high-dose Ara-C as postinduction therapy for AML.	[97]
NCT03379727, Interventional Phase IIIb	Midostaurin + "7+3" or "5+2" induction chemotherapy	To further assess the safety and efficacy of midostaurin plus chemotherapy in induction, consolidation, and maintenance monotherapy in young (≤60 years) and older (>60 years) ND-AML patients with FLT3-mutation.	CR/CRi	In this study, midostaurin in combination with intensive chemotherapy provided high response rates, irrespective of patient age, induction regimen ("7+3" or "5+2"), or the type of anthracycline used (daunorubicin or idarubicin) during the induction therapy.	[98]
ISRCTN-31682779, EudraCT-2013-00273021, Interventional Phase II/III	FLAG-Ida or DAC	To evaluate the survival benefit of chemotherapy intensification (FLAG-Ida or DAC) in older patients with AML who have not achieved an MRD–negative remission after a first course of DNR and Ara-C.	OS	In this study, older patients with AML considered fit and with evidence of residual disease after first induction, chemotherapy intensification improved survival. DAC intensification was better tolerated than FLAG-Ida.	[99]
NCT02283177, Interventional Phase II	Crenolanib in combination with intensive chemotherapy (Ara-C, DNR/IDA followed	To investigate the effects of crenolanib added to intensive chemotherapy on outcomes of ND-AML patients with FLT3-mutation.	CR/Cri<br>CIR<br>OS	In adults with ND-AML with FLT3-mutation, crenolanib plus intensive chemotherapy results in a high rate of deep responses and long-term survival with acceptable toxicity.	[100]

	by consolidation with Ara-C)				
NCT01246752, Interventional Phase III	AlloHCT vs. high-dose Ara-C for consolidation and salvage HCT only in case of relapse	To explore the optimal therapy for patients with intermediate-risk AML after first CR: alloHCT vs. standard consolidation chemotherapy	OS	In patients aged ≤60 years with intermediate-risk AML in first CR and an available donor, primary alloHCT did not confer superior OS compared with consolidation chemotherapy.	[101]

ALL

Clinical trial ID, study type, phase	Chemotherapeutic agent(s)	Objective	Primary endpoint	Conclusion	Ref
NCT03022747, Interventional Phase II	Oral 6-MP + allopurinol	To investigate the effects of adding allopurinol to 6-MP in ALL patients with TPMT wild-type patients without previous clinical signs of skewed 6-MP metabolism	e-TGN >200 nmol/mmol Hb	The addition of allopurinol to 6-MP shows promising outcomes, as it increased the levels of e-TGN while reducing the level of MeMP without adverse effects in ALL patients.	[102]
NCT00819351, Interventional Phase III	Oral 6-MP + MTX	To investigate the intensity of maintenance therapy as evaluated by MTX and 6-MP metabolite levels with the risk of symptomatic osteonecrosis in children and young adults with ALL.	Ery-TGN, Ery-MeMP, MTX polyglutamates, and DNA-TG	The intensity of maintenance therapy as measured by MTX and 6-MP metabolite levels was not associated with the risk of symptomatic osteonecrosis in children and young adults with ALL.	[103]
Retrospective study	IDA vs. L-DNR	To compare the efficacy and safety of IDA in comparison to L-DNR in combination with prednisone, VCR, and L-asparaginase in adults with HR-ALL.	OS, PFS, ORR	L-DNR was shown to be an effective drug within a multiagent approach, with a favourable overall profile, and with similar adverse events when compared with IDA in patients with HR-ALL.	[104]

NCT00846703, Interventional Phase IV	VCR and DEX pulses + 6-MP and MTX, vs. 6-MP and MTX only	To investigate the efficacy and safety of the addition of VCR/DEX pulses to conventional maintenance therapy and their applicability to the population in a large cohort of paediatric ALL who were treated with the modified BFM-2002 regimen.	EFS	Omitting nine pulses of VCR/DEX may reduce treatment burden and potentially improve quality of life in standard- to intermediate-risk patients. Conversely, in the HR-ALL cohort, incorporation of VCR/DEX pulses during the maintenance phase remains appropriate given the excellent outcomes observed.	[105]
ChiCTR1800014888, Interventional Phase IV	Decitabine	To investigate the efficacy and safety of low-dose decitabine on the prevention of adult ALL relapse after alloHSCT.	CIR, OS, DFS	Maintenance treatment with low-dose decitabine after alloHSCT may be used as a therapeutic option to reduce relapse in patients with adult ALL, especially in patients with T-ALL.	[106]

6-MP: 6-mercaptopurine; ALL: Acute lymphoblastic leukemia; AlloHCT/HSCT: Allogeneic hematopoietic cell transplantation/hematopoietic stem cell transplantation; AML: Acute myeloid leukemia; Ara-C: Cytarabine; CIR: Cumulative incidence of relapse; CR: Complete Remission; Cri: CR with Incomplete Count Recovery; DAC: Daunorubicin; Ara-C: Cladribine; DEX: Dexamethasone; DFS: Disease-free survival; DNA-TG: DNA-incorporated thioguanine nucleotides; DNR: Daunorubicin; EFS: Event-free survival; Ery-MeMP: Erythrocyte methylated 6-mercaptopurine metabolites; Ery-TGN: Erythrocyte thioguanine nucleotides; e-TGN: Erythrocyte levels of thioguanine nucleotides; FLAG-Ida: Fludarabine; ara-c; granulocyte colony-stimulating factor and IDA; FLT3: FMS-like tyrosine kinase 3; HDAC: High dose cytarabine; HR-ALL: High-risk ALL; IDA: Idarubicin; IDAC: Intermediate dose ara-C; L-DNR: Liposomal daunorubicin; MRD: Measurable residual disease; MTX: Methotrexate; ND-AML: Newly diagnosed-AML; ORR: Overall response rate; OS: Overall survival; PFS: Progression-free survival; TG: Thioguanine; TPMT: Thiopurine methyltransferase; VCR: Vincristine.

References

1. Jalte M, Abbassi M, El Mouhi H, Belghiti HD, Ahakoud M, Bekkari H. FLT3 mutations in acute myeloid leukemia: unraveling the molecular mechanisms and implications for targeted therapies. Cureus. 2023; 15(9): e45765. https://doi.org/10.7759/cureus.45765.
2. Kantarjian HM, DiNardo CD, Kadia TM, Daver NG, Altman JK, Stein EM, et al. Acute myeloid leukemia management and research in 2025. CA Cancer J Clin. 2025; 75(1): 46-67. https://doi.org/10.3322/caac.21873.
3. Padmakumar D, Chandraprabha VR, Gopinath P, Devi AR, Anitha GR, Sreelatha MM, et al. A concise review on the molecular genetics of acute myeloid leukemia. Leuk Res. 2021; 111: 106727. https://doi.org/10.1016/j.leukres.2021.106727.
4. Fedorov K, Maiti A, Konopleva M. Targeting FLT3 mutation in acute myeloid leukemia: current strategies and future directions. Cancers. 2023; 15(8):2312. https://doi.org/10.3390/cancers15082312.
5. Boddu PC, Kadia TM, Garcia-Manero G, Cortes J, Alfayez M, Borthakur G, et al. Validation of the 2017 European LeukemiaNet classification for acute myeloid leukemia with NPM1 and FLT3-internal tandem duplication genotypes. Cancer. 2019; 125(7):1091-100. https://doi.org/10.1002/cncr.31885.
6. Liu X, Ye Q, Zhao XP, Zhang PB, Li S, Li RQ, et al. RAS mutations in acute myeloid leukaemia patients: A review and meta-analysis. Clin Chim Acta. 2019; 489: 254-60. https://doi.org/10.1016/j.cca.2018.08.040.
7. Al-Kali A, Quintás-Cardama A, Luthra R, Bueso-Ramos C, Pierce S, Kadia T, et al. Prognostic impact of RAS mutations in patients with myelodysplastic syndrome. Am J Hematol. 2013; 88(5): 365-69. https://doi.org/10.1002/ajh.23410.
8. Bowen DT, Frew ME, Hills R, Gale RE, Wheatley K, Groves MJ, et al. RAS mutation in acute myeloid leukemia is associated with distinct cytogenetic subgroups but does not influence outcome in patients younger than 60 years. Blood. 2005; 106(6): 2113-19. https://doi.org/10.1182/blood-2005-03-0867.
9. Yohe S. Molecular genetic markers in acute myeloid leukemia. J Clin Med. 2015; 4(3): 460-78. https://doi.org/10.3390/jcm4030460.
10. Debnath A, Nath S. Prognosis and treatment in acute myeloid leukemia: a comprehensive review. Egypt J Med Hum Genet. 2024; 25(1): 91. https://doi.org/10.1186/s43042-024-00563-w.
11. Yang J, Zhao L, Wu Y, Niu T, Gong Y, Chen X, et al. The clinical features and prognostic implications of PTPN11 mutation in adult patients with acute myeloid leukemia in China. Cancer Medicine. 2023; 12(23):21111-17. https://doi.org/10.1002/cam4.6669.
12. Falini B, Brunetti L, Sportoletti P, Martelli MP. NPM1-mutated acute myeloid leukemia: from bench to bedside. Blood. 2020; 136(15):1707-21. https://doi.org/10.1182/blood.2019004226.
13. Gaidzik VI, Teleanu V, Papaemmanuil E, Weber D, Paschka P, Hahn J, et al. RUNX1 mutations in acute myeloid leukemia are associated with distinct clinico-pathologic and genetic features. Leukemia. 2016; 30(11):2160-68. https://doi.org/10.1038/leu.2016.126.
14. Schnittger S, Dicker F, Kern W, Wendland N, Sundermann J, Alpermann T, et al. RUNX1 mutations are frequent in de novo AML with noncomplex karyotype and confer an unfavorable prognosis. Blood. 2011; 117(8):2348-57. https://doi.org/10.1182/blood-2009-11-255976.
15. Döhner H, Estey E, Grimwade D, Amadori S, Appelbaum FR, Büchner T, et al. Diagnosis and management of AML in adults: 2017 ELN recommendations from an international expert panel. Blood. 2017; 129(4):424-47. https://doi.org/10.1182/blood-2016-08-733196.
16. Hou HA, Kuo YY, Liu CY, Chou WC, Lee MC, Chen CY, et al. DNMT3A mutations in acute myeloid leukemia: stability during disease evolution and clinical implications. Blood. 2012; 119(2): 559-68. https://doi.org/10.1182/blood-2011-07-369934.

17. Medeiros BC, Fathi AT, DiNardo CD, Pollyea DA, Chan SM, Swords R. Isocitrate dehydrogenase mutations in myeloid malignancies. Leukemia. 2017; 31(2): 272-81. https://doi.org/10.1038/leu.2016.275.
18. Patel JP, Gönen M, Figueroa ME, Fernandez H, Sun Z, Racevskis J, et al. Prognostic relevance of integrated genetic profiling in acute myeloid leukemia. N Engl J Med. 2012; 366(12): 1079-89. https://doi.org/10.1056/NEJMoa1112304.
19. Weissmann S, Alpermann T, Grossmann V, Kowarsch A, Nadarajah N, Eder C, et al. Landscape of TET2 mutations in acute myeloid leukemia. Leukemia. 2012; 26(5): 934-42. https://doi.org/10.1038/leu.2011.326.
20. Chou WC, Chou SC, Liu CY, Chen CY, Hou HA, Kuo YY, et al. TET2 mutation is an unfavorable prognostic factor in acute myeloid leukemia patients with intermediate-risk cytogenetics. Blood. 2011; 118(14): 3803-10. https://doi.org/10.1182/blood-2011-02-339747.
21. Tian X, Xu Y, Yin J, Tian H, Chen S, Wu D, et al. TET2 gene mutation is unfavorable prognostic factor in cytogenetically normal acute myeloid leukemia patients with NPM1+ and FLT3-ITD− mutations. Int J Hematol. 2014; 100(1): 96-104. https://doi.org/10.1007/s12185-014-1595-x.
22. Aslanyan MG, Kroeze LI, Langemeijer SM, Koorenhof-Scheele TN, Massop M, Van Hoogen P, et al. Clinical and biological impact of TET2 mutations and expression in younger adult AML patients treated within the EORTC/GIMEMA AML-12 clinical trial. Ann Hematol. 2014; 93(8): 1401-12. https://doi.org/10.1007/s00277-014-2055-7.
23. Ferrari A, Papayannidis C, Baldazzi C, Iacobucci I, Paolini S, Padella A, et al. Leukemia associated TP53 mutations in AML patients ARE strongly associated with complex karyotype and poor outcome. Blood. 2014; 124(21): 2379. https://doi.org/10.1182/blood.V124.21.2379.2379.
24. Niparuck P, Police P, Noikongdee P, Siriputtanapong K, Limsuwanachot N, Rerkamnuaychoke B, et al. TP53 mutation in newly diagnosed acute myeloid leukemia and myelodysplastic syndrome. Diagn Pathol. 2021; 16(1): 100. https://doi.org/10.1186/s13000-021-01162-8.
25. Hunter AM, Sallman DA. Current status and new treatment approaches in TP53 mutated AML. Best Pract Res Clin Haematol. 2019; 32(2): 134-44. https://doi.org/10.1016/j.beha.2019.05.004.
26. Krauth MT, Alpermann T, Bacher U, Eder C, Dicker F, Ulke M, et al. WT1 mutations are secondary events in AML, show varying frequencies and impact on prognosis between genetic subgroups. Leukemia. 2015; 29(3): 660-67. https://doi.org/10.1038/leu.2014.243.
27. Hou HA, Huang TC, Lin LI, Liu CY, Chen CY, Chou WC, et al. WT1 mutation in 470 adult patients with acute myeloid leukemia: stability during disease evolution and implication of its incorporation into a survival scoring system. Blood. 2010; 115(25): 5222-31. https://doi.org/10.1182/blood-2009-12-259390.
28. Boer JM, van der Veer A, Rizopoulos D, Fiocco M, Sonneveld E, de Groot-Kruseman HA, et al. Prognostic value of rare IKZF1 deletion in childhood B-cell precursor acute lymphoblastic leukemia: an international collaborative study. Leukemia. 2016; 30(1): 32-38. https://doi.org/10.1038/leu.2015.199.
29. Pan L, Chen Y, Weng K, Guo B, Zhuang S, Huang S, et al. Prognostic significance and treatment strategies for IKZF1 deletion in pediatric B-cell precursor acute lymphoblastic leukemia. BMC Cancer. 2024; 24(1): 1070. https://doi.org/10.1186/s12885-024-12828-z.
30. Zhang X, Rastogi P, Shah B, Zhang L. B lymphoblastic leukemia/lymphoma: new insights into genetics, molecular aberrations, subclassification and targeted therapy. Oncotarget. 2017; 8(39): 66728-41. https://doi.org/10.18632/oncotarget.19271.
31. Iacobucci I, Storlazzi CT, Cilloni D, Lonetti A, Ottaviani E, Soverini S, et al. Identification and molecular characterization of recurrent genomic deletions on 7p12 in the IKZF1 gene in a large cohort of BCR-ABL1–positive acute lymphoblastic leukemia patients: on behalf of Gruppo Italiano Malattie Ematologiche dell'Adulto Acute Leukemia Working Party (GIMEMA AL WP). Blood. 2009; 114(10): 2159-67. https://doi.org/10.1182/blood-2008-08-173963.

32. Mullighan CG, Su X, Zhang J, Radtke I, Phillips LA, Miller CB, et al. Deletion of IKZF1 and prognosis in acute lymphoblastic leukemia. N Engl J Med. 2009; 360(5): 470-80. https://doi.org/10.1056/NEJMoa0808253.
33. Fang Q, Song Y, Gong X, Wang J, Li Q, Liu K, et al. Gene deletions and prognostic values in B-linage acute lymphoblastic leukemia. Front Oncol. 2021; 11:677034. https://doi.org/10.3389/fonc.2021.677034.
34. Mullighan CG, Goorha S, Radtke I, Miller CB, Coustan-Smith E, Dalton JD, et al. Genome-wide analysis of genetic alterations in acute lymphoblastic leukaemia. Nature. 2007; 446(7137): 758-64. https://doi.org/10.1038/nature05690.
35. Fazio G, Bresolin S, Silvestri D, Quadri M, Saitta C, Vendramini E, et al. PAX5 fusion genes are frequent in poor risk childhood acute lymphoblastic leukaemia and can be targeted with BIBF1120. EBioMedicine. 2022; 83: 104224. https://doi.org/ 10.1016/j.ebiom.2022.104224.
36. Gu Z, Churchman ML, Roberts KG, Moore I, Zhou X, Nakitandwe J, et al. PAX5-driven subtypes of B-progenitor acute lymphoblastic leukemia. Nat Genet. 2019; 51(2): 296-307. https://doi.org/10.1038/s41588-018-0315-5.
37. Passet M, Boissel N, Sigaux F, Saillard C, Bargetzi M, Ba I, et al. PAX5 P80R mutation identifies a novel subtype of B-cell precursor acute lymphoblastic leukemia with favorable outcome. Blood. 2019; 133(3): 280-84. https://doi.org/10.1182/blood-2018-10-882142.
38. Auer F, Rüschendorf F, Gombert M, Husemann P, Ginzel S, Izraeli S, et al. Inherited susceptibility to pre B-ALL caused by germline transmission of PAX5 c. 547G> A. Leukemia. 2014; 28(5):1136-38. https://doi.org/10.1038/leu.2013.363.
39. Fouad FM, Eid JI. PAX5 fusion genes in acute lymphoblastic leukemia: A literature review. Medicine. 2023; 102(20): e33836. https://doi.org/10.1097/MD.0000000000033836.
40. Ha J, Kim B, Hahn S, Lee ST, Lyu CJ, Choi JR. A patient with B-cell acute lymphoblastic leukemia with PAX5-ETV6 rearrangement with dic (9; 12)(p13; p13) identified by chromosomal microarray. Ann Hematol. 2018; 97(8): 1505-07. https://doi.org/10.1007/s00277-018-3291-z.
41. Yu M, Al-Dallal S, Al-Haj L, Panjwani S, McCartney AS, Edwards SM, et al. Transcriptional regulation of the proto-oncogene Zfp521 by SPI1 (PU. 1) and HOXC13. Genesis. 2016; 54(10): 519-33. https://doi.org/10.1002/dvg.22963.
42. Mullighan CG, Zhang J, Kasper LH, Lerach S, Payne-Turner D, Phillips LA, et al. CREBBP mutations in relapsed acute lymphoblastic leukaemia. Nature. 2011; 471(7337): 235-39. https://doi.org/10.1038/nature09727.
43. Inthal A, Zeitlhofer P, Zeginigg M, Morak M, Grausenburger R, Fronkova E, et al. CREBBP HAT domain mutations prevail in relapse cases of high hyperdiploid childhood acute lymphoblastic leukemia. Leukemia. 2012; 26(8): 1797-803. https://doi.org/10.1038/leu.2012.60.
44. Dixon ZA, Nicholson L, Zeppetzauer M, Matheson E, Sinclair P, Harrison CJ, et al. CREBBP knockdown enhances RAS/RAF/MEK/ERK signaling in Ras pathway mutated acute lymphoblastic leukemia but does not modulate chemotherapeutic response. Haematologica. 2016; 102(4): 736-45. https://doi.org/10.3324/haematol.2016.145177.
45. Liu Y, Easton J, Shao Y, Maciaszek J, Wang Z, Wilkinson MR, et al. The genomic landscape of pediatric and young adult T-lineage acute lymphoblastic leukemia. Nat Genet. 2017; 49(8): 1211-18. https://doi.org/10.1038/ng.3909.
46. Clappier E, Collette S, Grardel N, Girard S, Suarez L, Brunie G, et al. NOTCH1 and FBXW7 mutations have a favorable impact on early response to treatment, but not on outcome, in children with T-cell acute lymphoblastic leukemia (T-ALL) treated on EORTC trials 58881 and 58951. Leukemia. 2010; 24(12): 2023-31. https://doi.org/10.1038/leu.2010.205.
47. Kox C, Zimmermann M, Stanulla M, Leible S, Schrappe M, Ludwig WD, et al. The favorable effect of activating NOTCH1 receptor mutations on long-term outcome in T-ALL patients

treated on the ALL–BFM 2000 protocol can be separated from FBXW7 loss of function. Leukemia. 2010; 24(12): 2005-13. https://doi.org/10.1038/leu.2010.203.
48. Weng AP, Ferrando AA, Lee W, Morris IV JP, Silverman LB, Sanchez-Irizarry C, et al. Activating mutations of NOTCH1 in human T cell acute lymphoblastic leukemia. Science. 2004; 306(5694): 269-71. https://doi.org/10.1126/science.1102160.
49. Heesch S, Goekbuget N, Stroux A, Tanchez JO, Schlee C, Burmeister T, et al. Prognostic implications of mutations and expression of the Wilms tumor 1 (WT1) gene in adult acute T-lymphoblastic leukemia. Haematologica. 2010; 95(6): 942-49. https://doi.org/10.3324/haematol.2009.016386.
50. Wang S, Wang C, Li T, Wang W, Hao Q, Xie X, et al. WT1 overexpression predicted good outcomes in adult B-cell acute lymphoblastic leukemia patients receiving chemotherapy. Hematology. 2020; 25(1): 118-24. https://doi.org/10.1080/16078454.2020.1735670.
51. Tosello V, Mansour MR, Barnes K, Paganin M, Sulis ML, Jenkinson S, et al. WT1 mutations in T-all. Blood. 2009; 114(5): 1038-45. https://doi.org/10.1182/blood-2008-12-192039.
52. Van Vlierberghe P, Palomero T, Khiabanian H, Van der Meulen J, Castillo M, Van Roy N, et al. PHF6 mutations in T-cell acute lymphoblastic leukemia. Nat Genet. 2010; 42(4): 338-42. https://doi.org/10.1038/ng.542.
53. Kurzer JH, Weinberg OK. PHF6 mutations in hematologic malignancies. Front Oncol. 2021; 11: 704471. https://doi.org/10.3389/fonc.2021.704471.
54. Todd MA, Picketts DJ. PHF6 interacts with the nucleosome remodeling and deacetylation (NuRD) complex. J Proteome Res. 2012; 11(8): 4326-37. https://doi.org/10.1021/pr3004369.
55. Van Vlierberghe P, Patel J, Abdel-Wahab O, Lobry C, Hedvat CV, Balbin M, et al. PHF6 mutations in adult acute myeloid leukemia. Leukemia. 2011; 25(1): 130-34. https://doi.org/ 10.1038/leu.2010.247.
56. Hsu YC, Chen TC, Lin CC, Yuan CT, Hsu CL, Hou HA, et al. Phf6-null hematopoietic stem cells have enhanced self-renewal capacity and oncogenic potentials. Blood Adv. 2019; 3(15): 2355-67. https://doi.org/10.1182/bloodadvances.2019000391.
57. Kubota Y, Terkawi L, Gurnari C, Bodo J, Kawashima N, Aly M, et al. Molecular and clinical PHF6 mutant myeloid neoplasia provides clues as to their pathogenesis and therapeutic targeting. Blood. 2022; 140(1): 8707–8708. https://doi.org/ 10.1182/blood-2022-169214.
58. Wang Q, Qiu H, Jiang H, Wu L, Dong S, Pan J, et al. Mutations of PHF6 are associated with mutations of NOTCH1, JAK1 and rearrangement of SET-NUP214 in T-cell acute lymphoblastic leukemia. Haematologica. 2011; 96(12): 1808-14. https://doi.org/10.3324/haematol.2011.043083.
59. Stone RM, Mandrekar SJ, Sanford BL, Laumann K, Geyer S, Bloomfield CD, et al. Midostaurin plus chemotherapy for acute myeloid leukemia with a FLT3 mutation. N Engl J Med. 2017; 377(5): 454-64. https://doi.org/10.1056/NEJMoa1614359.
60. [Internet] RYDAPT (midostaurin) for oral use. Silver Spring, MD: US Food and Drug Administration; 2017. https://www.accessdata.fda.gov/drugsatfda_docs/label/2017/207997s000lbl.pdf
61. [Internet] Umukoro C. The EMA grants midostaurin approval for the treatment of newly diagnosed FLT3+ AML patients. 2017. https://aml-hub.com/medical-information/the-ema-grants-rydapt-r-midostaurin-approval-for-the-treatment-of-newly-diagnosed-flt3-aml-patients
62. [Internet] Novartis drug Rydapt® (midostaurin) receives EU approval for newly diagnosed FLT3-mutated acute myeloid leukemia (AML) and three types of advanced systemic mastocytosis (SM). 2017. https://www.novartis.com/news/media-releases/novartis-drug-rydapt-midostaurin-receives-eu-approval-newly-diagnosed-flt3-mutated-acute-myeloid-leukemia-aml-and-three-types advanced-systemic-mastocytosis-sm
63. Erba HP, Montesinos P, Kim HJ, Patkowska E, Vrhovac R, Žák P, et al. Quizartinib plus chemotherapy in newly diagnosed patients with FLT3-internal-tandem-duplication-positive

acute myeloid leukaemia (QuANTUM-First): a randomised, double-blind, placebo-controlled, phase 3 trial. Lancet. 2023; 401(10388): 1571-83. https://doi.org/10.1016/S0140-6736(23)00464-6.

64. Short NJ, Daver N, Dinardo CD, Kadia T, Nasr LF, Macaron W, et al. Azacitidine, venetoclax, and gilteritinib in newly diagnosed and relapsed or refractory FLT3-mutated AML. J Clin Oncol. 2024; 42(13): 1499-508. https://doi.org/10.1200/JCO.23.01911.
65. Döhner H, Wei AH, Roboz GJ, Montesinos P, Thol FR, Ravandi F, et al. Prognostic impact of NPM1 and FLT3 mutations in patients with AML in first remission treated with oral azacitidine. Blood. 2022; 140(15): 1674-85. https://doi.org/10.1182/blood.2022016293.
66. Schlenk RF, Paschka P, Krzykalla J, Weber D, Kapp-Schwoerer S, Gaidzik VI, et al. Gemtuzumab ozogamicin in NPM1-mutated acute myeloid leukemia (AML): results from the prospective randomized AMLSG 09-09 phase-iii study. Blood. 2018; 132: 81. https://doi.org/10.1182/blood-2018-99-113442.
67. Montesinos P, Recher C, Vives S, Zarzycka E, Wang J, Bertani G, et al. Ivosidenib and azacitidine in IDH1-mutated acute myeloid leukemia. N Engl J Med. 2022; 386(16):1519-31. https://doi.org/10.1056/NEJMoa2117344.
68. Venugopal S, Takahashi K, Daver N, Maiti A, Borthakur G, Loghavi S, et al. Efficacy and safety of enasidenib and azacitidine combination in patients with IDH2 mutated acute myeloid leukemia and not eligible for intensive chemotherapy. Blood Cancer J. 2022; 12(1): 10. https://doi.org/10.1038/s41408-021-00604-2.
69. Metzeler KH, Walker A, Geyer S, Garzon R, Klisovic RB, Bloomfield CD, et al. DNMT3A mutations and response to the hypomethylating agent decitabine in acute myeloid leukemia. Leukemia. 2012; 26(5): 1106-07. https://doi.org/10.1038/leu.2011.342.
70. Yee K, Papayannidis C, Vey N, Dickinson MJ, Kelly KR, Assouline S, et al. Murine double minute 2 inhibition alone or with cytarabine in acute myeloid leukemia: results from an idasanutlin phase 1/1b study⋆. Leuk Res. 2021; 100: 106489. https://doi.org/10.1016/j.leukres.2020.106489.
71. Duong VH, Ruppert AS, Mims AS, Borate U, Stein EM, Baer MR, et al. Entospletinib with decitabine in acute myeloid leukemia with mutant TP53 or complex karyotype: A phase 2 substudy of the Beat AML Master Trial. Cancer. 2023; 129(15): 2308-20. https://doi.org/10.1002/cncr.34780.
72. Kadia TM, Cortes J, Ravandi F, Jabbour E, Konopleva M, Benton CB, et al. Phase II single-arm trial of cladribine and low-dose cytarabine alternating with decitabine as frontline therapy for older patients with acute myeloid leukemia. Lancet Haematol. 2018; 5(9): e411-21. https://doi.org/10.1016/S2352-3026(18)30132-7.
73. Garcia-Manero G, Goldberg AD, Winer ES, Altman JK, Fathi AT, Odenike O, et al. Eprenetapopt combined with venetoclax and azacitidine in TP53-mutated acute myeloid leukaemia: a phase 1, dose-finding and expansion study. Lancet Haematol. 2023; 10(4): e272-83. https://doi.org/10.1016/S2352-3026(22)00403-3.
74. Brown PA, Ji L, Xu X, Devidas M, Hogan LE, Borowitz MJ, et al. Effect of postreinduction therapy consolidation with blinatumomab vs chemotherapy on disease-free survival in children, adolescents, and young adults with first relapse of B-cell acute lymphoblastic leukemia: a randomized clinical trial. JAMA. 2021; 325(9): 833-42. https://doi.org/10.1001/jama.2021.0669.
75. Lew G, Chen Y, Lu X, Rheingold SR, Whitlock JA, Devidas M, et al. Outcomes after late bone marrow and very early central nervous system relapse of childhood B-acute lymphoblastic leukemia: a report from the Children's Oncology Group phase III study AALL0433. Haematologica. 2020; 106(1):46-55. https://doi.org/10.3324/haematol.2019.237230.
76. Goekbuget N, Schwartz S, Faul C, Topp MS, Subklewe M, Renzelmann A, et al. Dose reduced chemotherapy in sequence with blinatumomab for newly diagnosed older patients with Ph/BCR::

ABL negative B-precursor adult lymphoblastic leukemia (ALL): preliminary results of the GMALL Bold trial. Blood. 2023; 142:964.
77. Rowntree CJ, Kirkwood AA, Clifton-Hadley L, Farah N, Mansour MR, Hussain J, et al. First analysis of the UKALL14 randomized trial to determine whether the addition of nelarabine to standard chemotherapy improves event free survival in adults with T-cell acute lymphoblastic leukaemia (CRUK/09/006). Blood. 2021; 138:366. https://doi.org/10.1182/blood-2021-152355.
78. Senapati J, Kantarjian HM, Jain N, Short NJ, Kadia TM, Borthakur G, et al. ALL-78 venetoclax added to hyper-cvad-nelarabine and pegylated asparagine improves outcomes in patients with t-cell acute lymphoblastic leukemia/lymphoma. Clin Lymphoma Myeloma Leuk. 2024; 24: S283. https://doi.org/10.1016/S2152-2650(24)01137-6.
79. Stock W, Luger SM, Advani AS, Yin J, Harvey RC, Mullighan CG, et al. A pediatric regimen for older adolescents and young adults with acute lymphoblastic leukemia: results of CALGB 10403. Blood. 2019; 133(14): 1548-59. https://doi.org/10.1182/blood-2018-10-881961.
80. Borthakur G, Martinelli G, Raffoux E, Chevallier P, Chromik J, Lithio A, et al. Phase 1 study to evaluate Crenigacestat (LY3039478) in combination with dexamethasone in patients with T-cell acute lymphoblastic leukemia and lymphoma. Cancer. 2021; 127(3):372-80. https://doi.org/10.1002/cncr.33188.
81. Niu C, Wu D, Li AJ, Qin KH, Hu DA, Wang EJ, et al. Identification of a prognostic signature based on copy number variations (CNVs) and CNV-modulated gene expression in acute myeloid leukemia. Am J Transl Res. 2021; 13(12): 13683-96.
82. Han C, Zheng J, Li F, Guo W, Cai C. Novel prognostic signature for acute myeloid leukemia: bioinformatics analysis of combined CNV-driven and ferroptosis-related genes. Front Genet. 2022; 13: 849437. https://doi.org/10.3389/fgene.2022.849437.
83. Bolli N, Manes N, McKerrell T, Chi J, Park N, Gundem G, et al. Characterization of gene mutations and copy number changes in acute myeloid leukemia using a rapid target enrichment protocol. Haematologica. 2015; 100(2): 214-22. https://doi.org/10.3324/haematol.2014.113381.
84. Song Y, Fang Q, Mi Y. Prognostic significance of copy number variation in B-cell acute lymphoblastic leukemia. Front Oncol. 2022; 12: 981036. https://doi.org/10.3389/fonc.2022.981036.
85. Li M, Ye J, Chang M, Feng L, Liu T, Zhang D, et al. Polymorphisms in immunosuppression-related genes are associated with AML. Front Immunol. 2025; 16: 1530510. https://doi.org/10.3389/fimmu.2025.1530510.
86. Munir A, Akram AM, Jamil K, Tahir A. In Silico Post Translational Analysis of Functional Single Nucleotide Alterations in Human TERT Gene Associated with Acute Myeloid Leukemia. Pak Biomed J. 2023; 6(5): 24-32. https://doi.org/ 10.54393/pbmj.v6i05.881.
87. Tripon F, Bănescu C, Trifa AP, Crauciuc AG, Moldovan VG, Boglis A, et al. TERT rs2853669 as a predictor for overall survival in patients with acute myeloid leukaemia. Arch Med Sci. 2021; 18(1): 103-11. https://doi.org/10.5114/aoms/100673.
88. Tong Y, Xiang Y, Li B, Bao S, Zhou Y, Yuan W, et al. Association between TERT gene polymorphisms and acute myeloid leukemia susceptibility in a Chinese population: a case–control study. Cancer Cell Int. 2020; 20(1): 313. https://doi.org/10.1186/s12935-020-01335-3.
89. Sheng X, Tong N, Tao G, Luo D, Wang M, Fang Y, et al. TERT polymorphisms modify the risk of acute lymphoblastic leukemia in Chinese children. Carcinogenesis. 2013; 34(1): 228-35. https://doi.org/10.1093/carcin/bgs325.
90. Eskandari E, Hashemi M, Naderi M, Bahari G, Safdari V, Taheri M. Leukocyte telomere length shortening, hTERT genetic polymorphisms and risk of childhood acute lymphoblastic leukemia. Asian Pac J Cancer Prev. 2018; 19(6): 1515-21. https://doi.org/10.22034/APJCP.2018.19.6.1515.

91. Li M, Sun T, Chang M, Liu T, Feng L, Zhang D, et al. Impact of single nucleotide polymorphisms of immunomodulatory factors on treatment response and prognosis in acute myeloid leukemia. Front Immunol. 2025; 16: 1571332. https://doi.org/10.3389/fimmu.2025.1571332.
92. Thomas SM, Muruganantham JK, Sekar PK, Iyshwarya BK, Veerabathiran R. Investigating the impact of IKZF1 SNPs rs4132601 and rs11978267 on acute lymphoblastic leukemia: a comprehensive meta-analysis. J Egypt Natl Canc Inst. 2025; 37(1): 18. https://doi.org/10.1186/s43046-025-00274-2.
93. Alonaizan R, Alkhulaifi FM, Alomar S. Association of genetic variations in Toll-Like receptor 3 with Acute Lymphoblastic leukemia. J King Saud Univ Sci. 2024 Sep 1; 36(8): 103346. https://doi.org/10.1016/j.jksus.2024.103346.
94. Xabregas LA, Hanna FS, Magalhães-Gama F, Souza GL, Pereira DS, de Lima AB, et al. Association of Toll-like receptors polymorphisms with the risk of acute lymphoblastic leukemia in the Brazilian Amazon. Sci Rep. 2022; 12(1): 15159. https://doi.org/10.1038/s41598-022-19130-7.
95. Al-Absi B, Noor SM, Saif-Ali R, Salem SD, Ahmed RH, Razif MF, et al. Association of ARID5B gene variants with acute lymphoblastic leukemia in Yemeni children. Tumour Biol. 2017; 39(4): 1010428317697573. https://doi.org/10.1177/1010428317697573.
96. Zhou X, Liao F, Zhang J, Qin Y, Xu H, Ding Z, et al. Association of the independent polymorphisms in CDKN2A with susceptibility of acute lymphoblastic leukemia. Biosci Rep. 2018; 38(3): BSR20180331. https://doi.org/ 10.1042/BSR20180331.
97. Hunault M, Pautas C, Bertoli S, Dumas PY, Raffoux E, Hospital MA, et al. Intermediate-dose cytarabine as postinduction AML therapy. NEJM Evid. 2025; 4(7): EVIDoa2400326. https://doi.org/10.1056/EVIDoa2400326.
98. Sierra J, Montesinos P, Thomas X, Griskevicius L, Cluzeau T, Caillot D, et al. Midostaurin plus daunorubicin or idarubicin for young and older adults with FLT3-mutated AML: a phase 3b trial. Blood Adv. 2023 Nov; 7(21): 6441-50. https://doi.org/10.1182/bloodadvances.2023009847.
99. Russell NH, Thomas A, Hills RK, Thomas I, Gilkes A, Almuina NM, et al. Treatment intensification with either fludarabine, AraC, G-CSF and idarubicin, or cladribine plus daunorubicin and AraC on the basis of residual disease status in older patients with AML: Results from the NCRI AML18 trial. J Clin Oncol. 2025; 43(6): 694-704. https://doi.org/10.1200/JCO.24.00259.
100. Wang ES, Goldberg AD, Tallman M, Walter RB, Karanes C, Sandhu K, et al. Crenolanib and intensive chemotherapy in adults with newly diagnosed FLT3-mutated AML. J Clin Oncol. 2024; 42(15): 1776-87. https://doi.org/10.1200/JCO.23.01061.
101. Bornhäuser M, Schliemann C, Schetelig J, Röllig C, Kramer M, Glass B, et al. Allogeneic hematopoietic cell transplantation vs standard consolidation chemotherapy in patients with intermediate-risk acute myeloid leukemia: a randomized clinical trial. JAMA Oncol. 2023; 9(4): 519-26. https://doi.org/10.1001/jamaoncol.2022.7605.
102. Källström J, Niinimäki R, Fredlund J, Vogt H, Korhonen L, Castor A, et al. Effects of allopurinol on 6-mercaptopurine metabolism in unselected patients with pediatric acute lymphoblastic leukemia: a prospective phase II study. Haematologica. 2024; 109(9): 2846-53. https://doi.org/10.3324/haematol.2023.284390.
103. Toksvang LN, Andrés-Jensen L, Rank CU, Niinimäki R, Nersting J, Nielsen SN, et al. Maintenance therapy and risk of osteonecrosis in children and young adults with acute lymphoblastic leukemia: a NOPHO ALL2008 sub-study. Cancer Chemother Pharmacol. 2021; 88(5): 911-17. https://doi.org/10.1007/s00280-021-04316-z.
104. Zhang Q, Zhang CH, Wang ZD, Wang D. Efficacy and safety of induction chemotherapy with daunorubicin or idarubicin in the treatment of an adult with acute lymphoblastic leukemia. Tumori. 2022; 108(2): 182-88. https://doi.org/10.1177/03008916211032724.

105. Qiu KY, Wang JY, Huang LB, Li CG, Xu LH, Liu RY, et al. Vincristine and dexamethasone pulses in addition to maintenance therapy among pediatric acute lymphoblastic leukemia (GD-ALL-2008): An open-label, multicentre, randomized, phase III clinical trial. Am J Hematol. 2023; 98(6): 869-80. https://doi.org/10.1002/ajh.26910.

106. Liu J, Jiang ZX, Xie XS, Wan DM, Cao WJ, Wang M, et al. Maintenance treatment with low-dose decitabine after allogeneic hematopoietic cell transplantation in patients with adult acute lymphoblastic leukemia. Front Oncol. 2021; 11: 710545. https://doi.org/10.3389/fonc.2021.710545.
